# Kaposi's Sarcoma-Associated Herpesvirus Subversion of the Anti-Inflammatory Response in Human Skin Cells Reveals Correlates of Latency and Disease Pathogenesis

**DOI:** 10.1155/2014/246076

**Published:** 2014-02-17

**Authors:** Judith M. Fontana, Justin G. Mygatt, Katelyn L. Conant, Chris H. Parsons, Johnan A. R. Kaleeba

**Affiliations:** ^1^Department of Microbiology and Immunology, Uniformed Services University of the Health Sciences, 4301 Jones Bridge Road, Bethesda, MD 20814, USA; ^2^Department of Medicine and Microbiology, Stanley S. Scott Cancer Center, Louisiana State University Health Science Center, New Orleans, LA 70112, USA

## Abstract

KSHV is the etiologic agent for Kaposi's sarcoma (KS), a neoplasm that manifests most aggressively as multifocal lesions on parts of human skin with a propensity for inflammatory reactivity. However, mechanisms that control evolution of KS from a benign hyperplasia to the histologically complex cutaneous lesion remain unknown. In this study, we found that KSHV induces proteomic and morphological changes in melanocytes and melanoma-derived cell lines, accompanied by deregulation of the endogenous anti-inflammatory responses anchored by the MC1-R/**α**-MSH signaling axis. We also identified two skin-derived cell lines that displayed differences in ability to support long-term KSHV infection and mapped this dichotomy to differences in (a) NF-**κ**B activation status, (b) processing and expression of KSHV latency-associated nuclear antigen isoforms putatively associated with the viral lytic cycle, and (c) susceptibility to virus-induced changes in expression of key anti-inflammatory response genes that antagonize NF-**κ**B, including MC1-R, POMC, TRP-1, and xCT. Viral subversion of molecules that control the balance between latency and lytic replication represents a novel correlate of KSHV pathogenesis and tropism in skin and underscores the potential benefit of harnessing the endogenous anti-inflammatory processes as a therapeutic option for attenuating cutaneous KS and other proinflammatory outcomes of KSHV infection in high-risk individuals.

## 1. Introduction

Kaposi's sarcoma (KS) is a highly angioproliferative neoplasm etiologically linked to infection with Kaposi's sarcoma-associated herpesvirus (KSHV), also formally known as human herpesvirus 8 (HHV-8) [1, 2]. Four epidemiological forms of KS have been described and distinguished based on age, sex, geographical location, socioeconomic status, previous exposure to parasitic infections, coinfection with HIV, and the extent of iatrogenic organ transplant-associated immune suppression [3–5]. Although the precise trigger(s) for KS are currently not known, all forms of the lesion display histopathological characteristics consistent with a shared requirement for chronic inflammation, leading to the notion that a preexisting or virus-induced state of inflammation is essential for creating the physiologic conditions necessary for KS development and progression. Indeed, at the early patch stage, the lesion produces and/or paracrinally responds to pro-inflammatory cytokines and growth factors including interferon-*γ* (IFN-*γ*), tumor necrosis factor-*α* (TNF-*α*), interleukin-1*β* (IL-1*β*), IL-2, and IL-6 [6–9] that support expression of adhesion molecules and chemokines that mediate recruitment of circulating monocytes to the lesion. The early stage is also associated with secretion of protumorigenic mediators such as vascular endothelial growth factor (VEGF) that drives the angiogenic increase in hyperproliferating spindle cells, which in turn form the basic slit-like framework for erythrocyte extravasation, neovascularization, and plasma cell infiltration; together, these biologic processes are believed to contribute to the complex cellularity found in KS lesions [6, 7, 10–14].

Generally, expression of pro-inflammatory proteins within and around the KS lesion is primarily controlled by the nuclear factor-*κ*-B (NF-*κ*B) transcription factor, an important mediator of inducible gene expression during cellular growth, immune reactivity, and pathological inflammation [15]. Although the list of molecules that comprise the definitive NF-*κ*B signaling axis is rapidly expanding [16], the classical transactivating form of NF-*κ*B is comprisesd of RelA (p65)/p50 heterodimers which, in unstimulated cells, are sequestered in an inactive form in the cytoplasm via physical association with one of several inhibitors, designated I*κ*B [15]. Upon extracellular stimulation, signal transduction events rapidly lead to activation of the I*κ*B kinase (IKK) complex composed of two catalytic subunits (IKK*α* and *β*) and a regulatory “NF-*κ*B essential modulator subunit” (NEMO). Activated IKK (mainly IKK*β*) phosphorylates I*κ*B*α*, triggering its polyubiquitination and proteasomal degradation; this liberates the active NF-*κ*B homo- or heterodimers, which subsequently translocate to the nucleus where they drive transcription of NF-*κ*B-responsive genes [15].

The role of NF-*κ*B in KSHV pathogenesis was brought into focus by the observation that sustained, virus-induced activation of NF-*κ*B following infection is required not only for establishment and maintenance of latency [17, 18] but also for oncogenic transformation [19, 20]. Accordingly, KSHV commits a large portion of its coding potential towards NF-*κ*B activation, primarily via the functions of the viral FADD-like interleukin-1-*β*-converting enzyme [FLICE/caspase-8]-inhibitory protein (vFLIP) [21], the viral G-protein-coupled receptor (vGPCR) [22, 23], K1 [24], K13 [25], K15 [26], the viral tegument protein encoded by open reading frame-75 [25], as well as a cluster of microRNAs, many of which are linked to regulation of immune reactivity, cell survival, and the tumorigenic potential of KSHV [27–29]. Ironically, NF-*κ*B activation is associated with viral latency, whereas its inhibition results in viral entry into the lytic cycle [17, 18]. It was recently reported, for example, that NF-*κ*B not only activates expression of KSHV latent genes including LANA, v-FLIP, and v-Cyclin, but also negatively regulates the transactivating potential of the KSHV major lytic switch regulator, RTA [17, 30, 31]. NF-*κ*B inhibits RTA recruitment to viral promoters either through direct competition with RBP-J*κ* (which cooperatively enhances RTA transactivation by binding DNA adjacent to low-affinity RTA-binding sites within RTA-responsive genes [32–34]) or by sequestering RTA from RBP-J*κ*, both of which were shown to depend on the relative amount and activation status of NF-*κ*B [35].

Clearly, the pro-inflammatory function of NF-*κ*B controls the balance between viral latency and lytic replication by regulating accessibility of the KSHV genome to transactivating factors, and is therefore a fundamental determinant of KS development in skin and other target sites. Given this regulatory control, it is intriguing that the host anti-inflammatory mechanisms that are normally activated to attenuate inflammation in skin somehow fail to control disease induction in this target organ. To explore the molecular basis for this paradoxical outcome, we tested the hypothesis that KSHV infects and subverts the local anti-inflammatory processes of skin cells so that they are unable to overcome the virus-induced pro-inflammatory reactions that support KS histogenesis. We now present the first evidence that melanocytes can be infected by KSHV and in turn may be co-opted as an important cellular determinant of KSHV pathogenesis in skin. Importantly, we have identified two cell lines that displayed dichotomous properties with respect to their ability to support the KSHV latency program and mapped these cytologic attributes to (a) differences in the state of virus-induced NF-*κ*B activation immediately following primary infection and upon establishment of latency, (b) expression of unique LANA isoforms associated with lytic replication, and (c) susceptibility of persistently infected cells to viral impairment of key components of the endogenous anti-inflammatory response pathways. Together, our findings represent the first delineation of the important role that skin cells may play in KSHV pathogenesis and reveal both the cellular correlates for maintenance of viral latency and the nature of interactions between KSHV and the host anti-inflammatory axis that could serve as predictive markers for cutaneous KS.

## 2. Materials and Methods

### 2.1. Cell Culture

Vero cells (ATCC; Manassas, VA) and rKSHV.219-infected Vero cells (a kind gift from Jeffrey Vieira, University of Washington, Seattle, WA) were cultured in DMEM (Quality Biological, Inc., Gaithersburg, MD) supplemented with 10% fetal bovine serum (FBS; HyClone Laboratories, Logan, UT) and puromycin (10 *μ*g/mL; Calbiochem, EMD Chemicals, Inc., Gibbstown, NJ). As described previously [36], rKSHV.219 expresses enhanced green fluorescent protein (GFP) under the control of the strong cellular elongation factor 1-*α* promoter and the Ds-red fluorescent protein (RFP) from the KSHV lytic polyadenylated nuclear (PAN) RNA promoter, in addition to the gene for puromycin resistance as a selectable marker for maintenance of the viral episome in rKSHV.219-infected cells [36]. Normal human adult epidermal primary melanocytes (NHEM-Ad; Lonza Walkersville, Inc., Walkersville, MD) were cultured in 254CF media supplemented with 0.1 mM CaCl_2_ and human melanocyte growth supplement with PMA (Cascade Biologics, Invitrogen, Carlsbad, CA). MeWo, a highly-pigmented cell line derived from a nodular lymph node metastasis in a patient with malignant melanoma [37], was obtained from ATCC and cultured in EMEM (Quality Biological, Inc.) supplemented with 10% FBS. Mel1700, a benign human melanoma-derived cell line, was provided by Maurice Zauderer (Vaccinex, Inc., Rochester, NY) and cultured in RPMI-1640 (Quality Biological, Inc.) supplemented with 20% FBS. rKSHV.219-infected MeWo and Mel1700 cells were derived in our laboratory and maintained under selection with puromycin at concentrations of 0.5 *μ*g/mL and 1 *μ*g/mL, respectively. The immortalized human keratinocyte cell line, HaCat [38], was cultured in DMEM supplemented with 10% FBS. rKSHV.219-infected HaCat cells were also maintained in DMEM supplemented with 10% FBS and puromycin (0.5 *μ*g/mL). The body cavity-based lymphoma cell line, BCBL-1, that is persistently infected with KSHV, was obtained from Michael McGrath and Don Ganem via the NIH AIDS Research and Reference Reagent Program, Division of AIDS (NIAID, NIH, Bethesda, MD), and was cultured in RPMI-1640 supplemented with 10% FBS.

### 2.2. Virus Infections

For infection studies, cultures of rKSHV.219-infected Vero cells were induced with 6 mM sodium *n*-butyrate (NaB; Sigma, St. Louis, MO) and monitored for expression of RFP. Four days after induction, cells were scraped, resuspended in spent media, and collected by centrifugation at 4°C, as previously described [36]. The supernatant containing concentrated KSHV particles was maintained on ice while the cell pellet was resuspended in 2 mLs of DMEM and frozen and thawed two times to release cell-associated viral particles. The resulting lysate was centrifuged at 2500 rpm for 10 minutes at 4°C to remove debris and the clarified supernatant was combined with the original culture supernatant, sterile-filtered through a 0.45 *μ*m membrane filter, and stored at −80°C. Uninfected Vero cells were treated with 6 mM NaB and harvested in parallel to be used for mock infection.

To generate chronically rKSHV.219-infected cells, uninfected cells were plated in T-25 flasks in their respective growth media supplemented with 10% FBS. At 70% confluency, media were removed from each flask and 2 mLs of the Vero-derived rKSHV.219 supernatant was added. After one hour of incubation at 37°C, 3 mLs of media (with 5% FBS) was added and further incubated at 37°C and monitored for the expression of GFP. Subsequently, a progressively increasing concentration (0.1–10 *μ*g) of puromycin/mL was added to the culture in order to enrich for rKSHV.219-infected cells over time. Mock infections were performed in parallel using uninfected Vero cell supernatant and cells were harvested for further analysis at the time of puromycin addition for rKSHV.219-infected cells. For infection of HaCat cells, a T-75 flask of rKSHV.219-infected Mel1700 cells was induced with 6 mM NaB for two days to achieve optimal amounts of RFP positive cells (reactivation conditions were determined empirically). Cell supernatants containing reactivated KSHV were collected, spun at 2500 rpm for 10 minutes at 4°C to remove floating cells and other debris, sterile-filtered through a 0.45 *μ*m membrane filter, and added to uninfected HaCat cells. HaCat cells infected with Mel1700-derived rKSHV.219 were monitored for the appearance of GFP and selected with puromycin (0.5 *μ*g/mL starting at day 3 postinfection).

### 2.3. Antibodies and Reagents

The following antibodies were purchased from Santa Cruz Biotechnology, Inc. (Santa Cruz, CA): polyclonal anti-GAPDH (L-20), monoclonal anti-TRP-1 (H-90), anti-Melan A (FL-118), anti-MC1R (H-60), and HRP-labeled goat anti-rat secondary antibody. Rat anti-LANA monoclonal antibody LN35 was purchased from Abcam (Cambridge, MA). AlexaFluor-647-labeled chicken anti-rat IgG secondary antibody (Cat no. A21472) and AlexaFluor-568-labeled goat anti-rat IgG secondary antibody (Cat no. A11077) were purchased from Molecular Probes (Invitrogen, Carlsbad, CA). Unless otherwise indicated, all rabbit monoclonal antibodies against key components of the NF-*κ*B pathway, as well as a polyclonal rabbit antibody to phosphorylated NF-*κ*B p65, were purchased from Cell Signaling Technology (Danvers, MA). Additional antibodies to total NF-*κ*B p65 were purchased from Biosource International (Camarillo, CA) and used in western blot assays with HRP-labeled sheep anti-mouse (Amersham Biosciences, GE Healthcare Life Sciences, Piscataway, NJ) or HRP-labeled donkey anti-rabbit (Chemicon, Millipore, Billerica, MA) secondary antibodies, respectively. Western blot analysis or human xCT was carried out using a previously described polyclonal rabbit antibody to the N-terminal peptide of human SLC7A11 (xCT) [39], or with polyclonal anti-xCT antibody NB300-318 from Novus Biologicals, (Littleton, CO). Recombinant alpha-MSH was purchased from EMD-Calbiochem (La Jolla, California).

### 2.4. Microscopy

Images were captured using an AxioCam MRm digital camera (Carl Zeiss, Inc.) attached to a Zeiss Axio Observer.A1 inverted fluorescent microscope (Carl Zeiss, Inc.). Pseudocoloring and other image analyses were performed using AxioVision Release 4.6 software.

### 2.5. Flow Cytometry

Cells were washed, trypsinized, resuspended in FACS Buffer (sterile filtered 1% FBS in PBS without Ca^2+^ or Mg^2+^), and counted. 1 × 10^6^ cells were fixed with 2% paraformaldehyde (Electron Microscopy Sciences, Fort Washington, PA) and transferred into 12 × 75 FACS tubes (Falcon, #352063) for analysis by flow cytometry. Cell measurements were acquired using the BD Biosciences LSRII Cell Analyzer and FACSDiva software. Data was analyzed using FlowJo software (version 8.7.3).

### 2.6. PCR Amplification

Total DNA was extracted from 5 × 10^5^ rKSHV.219-infected or uninfected cells using the DNeasy Blood and Tissue Kit (Qiagen, Valencia, CA), and PCR amplification was performed with Platinum PCR SuperMix High Fidelity (Invitrogen), as recommended by the manufacturer. For detection of viral DNA by PCR, the KS330 primer pair derived from KSHV ORF26 (Table S1) was used to amplify a 232 bp (nt 987–1218) fragment using the following conditions: 94°C for two minutes, then 35 cycles of 94°C for one minute, 58°C for one minute, and 72°C for one minute, followed by 72°C for five minutes, as previously described [1]. Samples were analyzed by gel electrophoresis on 2% agarose gels stained with ethidium bromide.

### 2.7. RT-PCR

Total RNA was extracted from cell pellets using Trizol Reagent (Invitrogen), digested with the RNase-free DNase set (Qiagen) and quantified using a ND-1000 NanoDrop spectrophotometer supported by the associated software, version 3.1.2. RT-PCR was performed on 0.5 *μ*g DNase-digested RNA using the SuperScript One-Step RT-PCR with Platinum Taq kit (Invitrogen) with primer sets specific for viral or cellular genes (Table S1). Amplification products were analyzed by gel electrophoresis on 1% agarose gels stained with ethidium bromide.

### 2.8. Immunofluorescence

Cells were seeded in eight-well chamber slides (Lab-Tek Chamber Slide System, Nalge Nunc) at a concentration of 1 × 10^5^ cells per well and allowed to adhere overnight at 37°C. For direct immunofluorescence, the cells were fixed the next day in 2% paraformaldehyde (Electron Microscopy Sciences, Fort Washington, PA) in PBS for 30 minutes and then permeabilized in 2% paraformaldehyde and 0.1% Triton X-100 (Calbiochem, La Jolla, CA) in PBS for an additional 30 minutes. After fixation, the cells were washed once with PBS and incubated in blocking buffer (2% FBS in PBS) for one hour at 37°C. Blocking buffer was then removed and the cells were further incubated with anti-LANA primary antibodies in fresh blocking buffer for one hour at 37°C. Cells were washed five times with PBS and then incubated with fluorescently-labeled secondary antibodies for one hour at 37°C. After five washes with PBS to remove unbound antibody, coverslips were mounted onto the slides using Vectashield Mounting Medium for Fluorescence with DAPI (Vector Laboratories, Burlingame, CA) and cells were visualized using fluorescence microscopy.

### 2.9. Western Blot

Cell pellets (1 × 10^6^ cells per pellet) were lysed in RIPA buffer (0.1% SDS, 1% NP-40, 150 mM NaCl, 1 mM EDTA, 50 mM Tris-HCl pH 7.5, 0.5% deoxycholate, 50 *μ*g/mL BSA) and electrophoresed under reducing conditions on a NuPAGE 4–12% Bis-Tris gel with MES SDS Running Buffer (Invitrogen). Resolved proteins were transferred from the gel to a PVDF membrane (Invitrogen) and blocked overnight in PBS containing 5% nonfat powdered milk and 0.1% Tween-20. Primary and secondary antibodies were each diluted in blocking buffer and incubated with the blots for one hour, rocking at room temperature. Blots were washed three times (0.05% NP-40 in PBS) for 15 minutes after incubation with each antibody and developed using SuperSignal West Femto substrate (Thermo Scientific, Rockford, IL).

## 3. Results

### 3.1. Human Melanocytes Can Be Infected by KSHV

**Figure 1:**
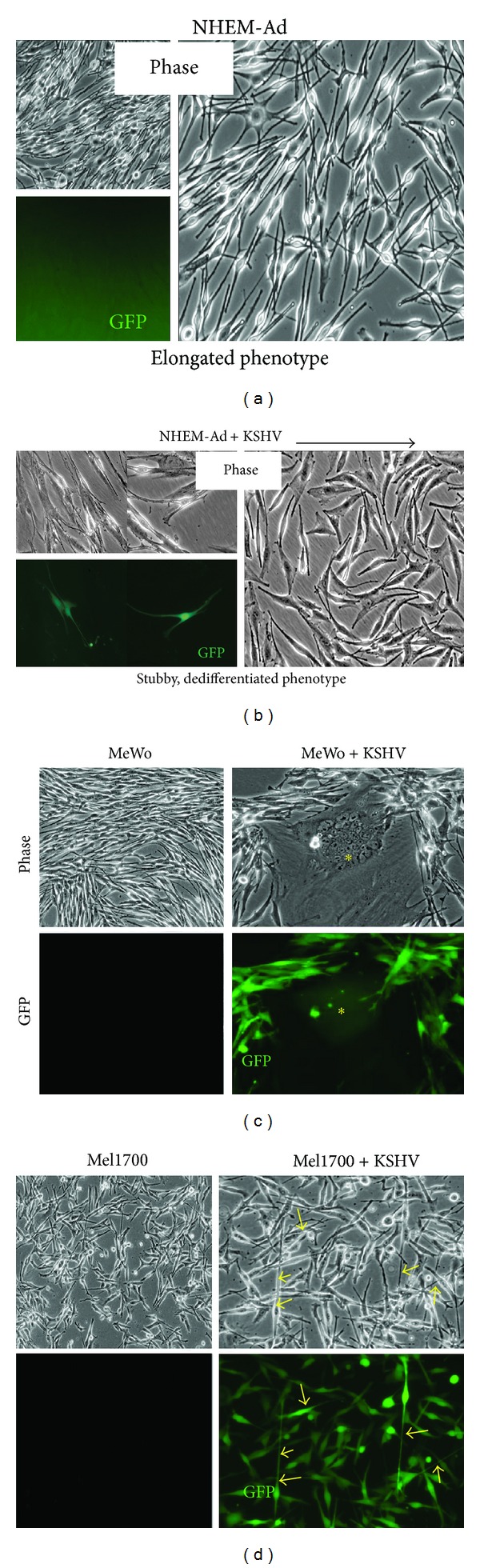
KSHV infection of primary melanocytes and melanoma-derived cells is associated with virus-induced morphological changes. (a) (b): Representative phase and GFP images of uninfected (a) or rKSHV.219-infected (b) primary NHEM-Ad melanocytes at day two after infection. Rightmost panels are enlarged (20x) phase contrast views of uninfected and infected cultures, respectively. (c) (d): Representative images of uninfected or rKSHV.219-infected MeWo cells (c) and Mel1700 cells (d). Multinucleated cells in infected MeWo cells are denoted with asterisks, whereas yellow arrows in infected Mel1700 cells point to elongated dendritic spines putatively induced by the virus.

We determined the susceptibility of skin cells to KSHV by innoculating primary adult melanocytes (NHEM-Ad) and two melanoma-derived cell lines (MeWo and Mel1700) with rKSHV.219 and then analyzed: (i) postentry expression of virus-encoded GFP, (ii) viral genome content by PCR, (iii) viral gene expression by RT-PCR, and (iv) productive release of infectious virus upon lytic reactivation. Compared to controls, infected melanocytes (NHEM-Ad) and MeWo cells lost their elongated morphology and acquired a stubby, dedifferentiated phenotype (Figures 1(b) and 1(c)). Persistently infected MeWo cells also formed large, multinucleated syncytia (Figures 1(c) and S1A; yellow asterisks), possibly induced by viral glycoprotein-mediated cell fusion [40]. On the other hand, persistently infected Mel1700 cells did not become stubby but instead expressed outgrowths of elongated dendritic spines (Figures 1(d) and S1C; arrows). These morphologic changes are reminiscent of the neuroendocrine-like features characteristically associated with subskin lesions [41–43] and may be the first indication of hitherto unexplored aspects of virus-host interactions that contribute to pathology in skin. After a series of a selective passage of infected cells in the presence of puromycin, we were able to generate long-term cultures (almost 100% infection rate) of persistently infected KSHV-infected cells (see Figure S1 in Supplementary Material available online at http://dx.doi.org/10.1155/2014/246076), allowing us to further characterize the virologic outcomes of KSHV infection in these cells.

### 3.2. Differential Induction of the KSHV Lytic Cycle in Skin Cells Suggests Cell Line-Specific Correlates of Viral Tropism

**Figure 2:**
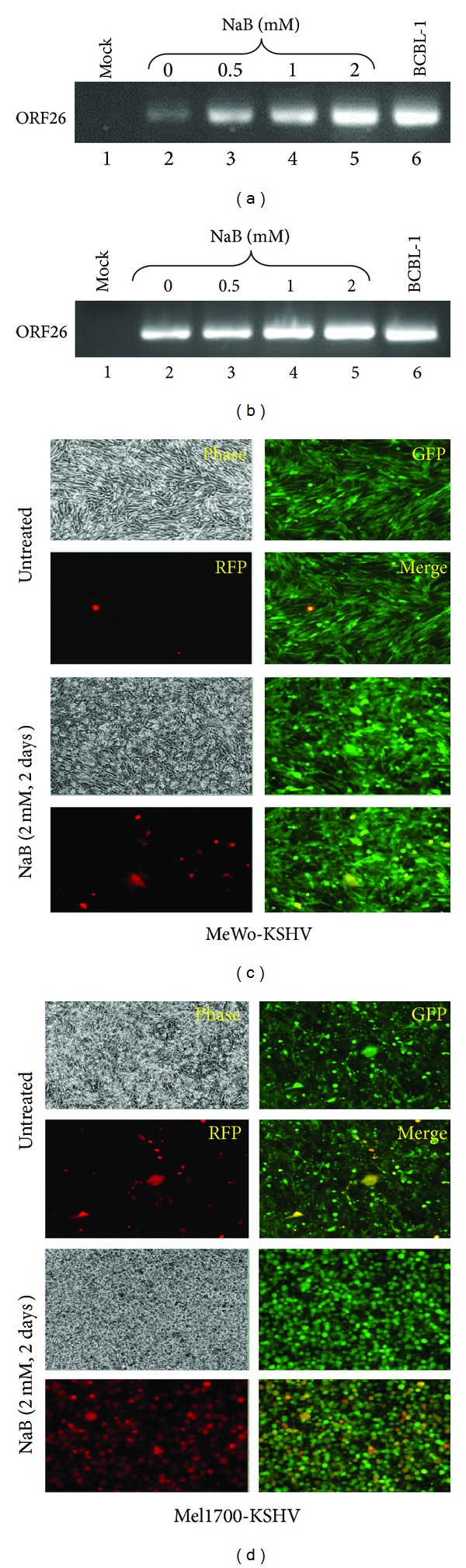
Differential induction of viral lytic cycle in skin cells reveals cell line-specific correlates of latency. (a) and (b): Total DNA was extracted from 5 × 10^5^ rKSHV.219-infected MeWo-KSHV cells (a) or Mel1700-KSHV cells (b) either mock-treated or treated with increasing concentrations of NaB, as indicated. An equal amount of normalized viral DNA was then used for PCR amplification of the virus genome using ORF26-specific primers (Table S1). Approximately 10 ng of DNA from KSHV-infected BCBL-1 cells was used as a positive control. (c) and (d): Representative phase, GFP, RFP, and color-merged images of infected MeWo-KSHV (c) or Mel1700-KSHV cells (d) either untreated or treated with 2 mM NaB for two days.

Long-term infection of skin cells was confirmed by PCR amplification of viral DNA isolated from cells treated with increasing amounts of *n*-butyrate (NaB), a histone deacetylase inhibitor known to induce viral reactivation [44]. As shown in Figure 2, the template from uninduced Mel1700-KSHV cells produced a more intense viral DNA product compared to the target template from uninduced MeWo-KSHV cells (Figures 2(a) and 2(b); compare lane 2 in each case), implying that Mel1700-KSHV cells may harbor more viral genome copies than MeWo-KSHV cells, which we confirmed by dilutional analysis against preset standards of viral DNA from KSHV^+^ BCBL-1 cells previously determined to contain approximately 100 genome copies per cell [45]. To determine whether the putative difference in viral genome content reflected a difference in the replicative potential of the virus in these cells, we used epifluorescence to analyze spontaneous or NaB-induced virus reactivation based on expression of RFP, which is transcribed under the control of a strictly lytic viral PAN promoter [36]. As shown in Figure 2(c), RFP was virtually undetectable in untreated MeWo-KSHV cells, but increased slightly after treatment with 2 mM NaB. In contrast, the already high level of spontaneous RFP expression in Mel1700-KSHV cells was further increased by NaB (Figure 2(d)), further suggesting that Mel1700 cells may have a higher propensity for supporting viral lytic reactivation. However, we were mindful of the fact that differences in cell density could also influence the timing and/or robustness of viral reactivation in confluent cultures of infected cells, so we used flow cytometry to more accurately quantitate the time-dependent increase in RFP+ cells following NaB treatment. As shown in Figure S2A, approximately 0.9% of untreated MeWo-KSHV cells were RFP+, compared to 7.9% of untreated Mel1700-KSHV. At each time point after NaB treatment, the number of RFP+ cells in MeWo and Mel1700 cells increased to approximately 30% versus 36% after 1 day, 41% versus 45% after 2 days, and to 46% versus 57% after 2 days, respectively. This temporal change in RFP expression is also illustrated in various formats (Figures S2B–S2D), all of which reveal that Mel1700 cells support a higher level of spontaneous and/or drug-induced KSHV reactivation than MeWo cells. Furthermore, virions from the highly inducible Mel1700-KSHV cells were infectious for human HaCat keratinocytes, which subsequently maintained GFP expression (Figure S3A) and viral DNA after more than 48 days of continuous culture in presence of puromycin (Figure S3B). Moreover, infected HaCat-KSHV cells could also support viral lytic replication, as evidenced by foci of RFP-expressing cells upon treatment with a low dose of NaB (Figure S3C).

### 3.3. KSHV-Infected Skin Cells Support the Full Spectrum of Viral Gene Expression

**Figure 3:**
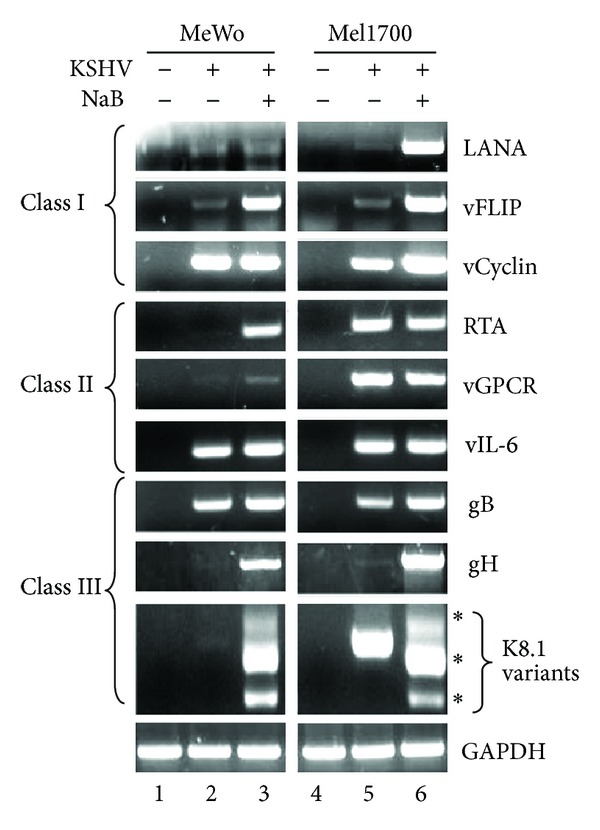
rKSHV.219-infected melanoma cells support the full spectrum of latent and lytic viral gene expression. Total RNA from mock (−) or rKSHV.219-infected (+) MeWo and Mel1700 cells either left untreated (−) or induced (+) with 2 mM NaB for two days was used as template for RT-PCR amplification of viral transcripts using primer sets specific for genes belonging to the latent class I (LANA, vFLIP, vCyclin), latent/inducible class II (RTA, vGPCR, vIL6), or the strictly lytic class III (gB, gH, K8.1) (Table S1). GAPDH was used as a template loading control.

To determine whether differences in viral lytic replication in MeWo and Mel1700 cells (Figure 2) also reflected patterns of viral gene expression in these cells, we analyzed the transcriptional profile of selected viral genes belonging to each of the three previously described classes of viral transcription in KSHV-infected cells [46–48]. Thus, class I genes include LANA, v-FLIP, and v-Cyclin that are constitutively expressed, while class II genes include the immediate early master switch regulator of the KSHV viral lytic cycle, RTA, as well as Orf74 (v-GPCR), and K2 (v-IL-6), which are constitutively expressed but highly inducible regulators of viral gene expression and the host microenvironment. On the other hand, class III genes include strictly lytic genes such as gB, gH, and K8.1 that encode viral structural proteins. Viral gene transcription was assessed by semiquantitative RT-PCR; all RNA samples were treated with DNase I prior to the RT-PCR reaction, and as a consequence no amplification product was detected in a pre-RT-PCR control experiment in which *Reverse-Transcriptase* (RT) was omitted from the reactions (Figure S4A). In addition, no viral DNA was detected in DNase I-treated RNA samples (Figure S4B), confirming that we had successfully removed contaminating viral DNA. As shown in Figure 3, all genes tested were expressed in both cell lines, especially following NaB treatment. However, an important distinction was evident in the expression of key markers of stage-specific replication, most notably the immediate early RTA, the early/late vGPCR, and the strictly late K8.1. While these transcripts were expressed only in NaB-treated (but not in uninduced) MeWo-KSHV cells, they were abundantly expressed in untreated Mel1700-KSHV cells (Figure 3, compare lanes 2 and 5). Given that RTA transactivates the promoters of several lytic KSHV genes including its own [46–48], the difference in RTA expression in the absence of drug induction could explain the higher level of spontaneous viral reactivation and virion output in infected Mel1700-KSHV cells compared to their MeWo-KSHV counterparts.

### 3.4. Differential Expression of LANA in KSHV-Infected Cells Highlights Diffuse Nuclear LANA Expression as a Marker of Viral Lytic Replication

**Figure 4:**
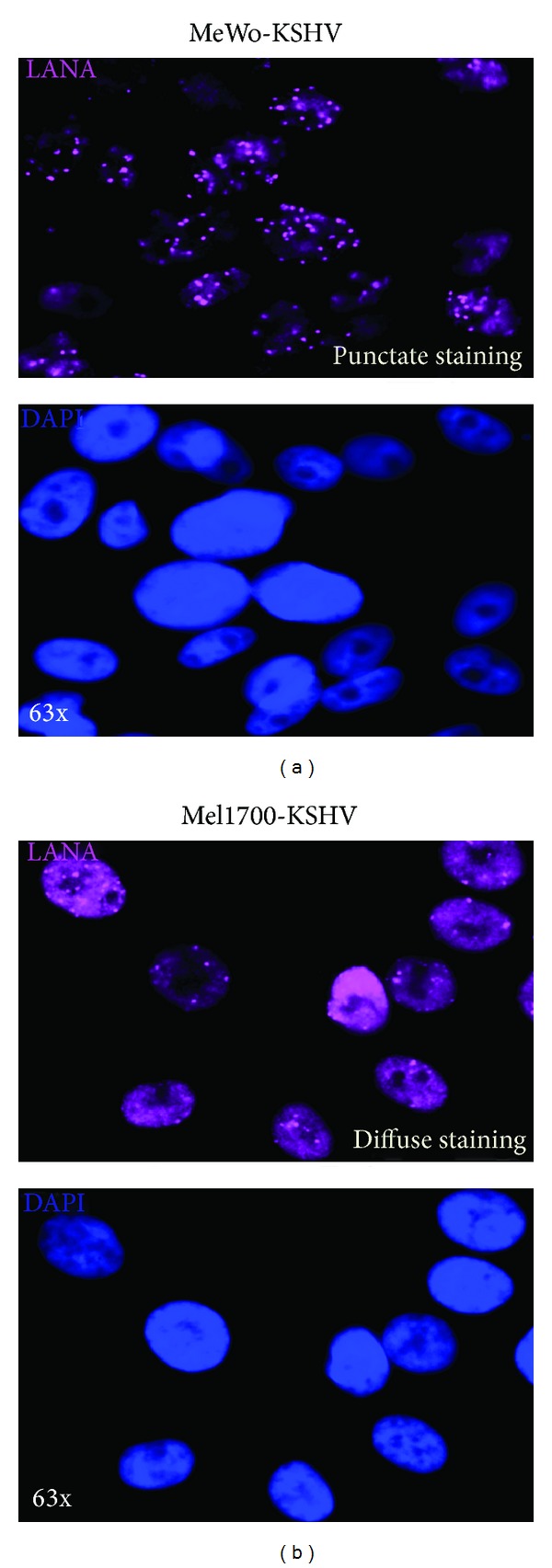
Differential expression of LANA in KSHV-infected MeWo and Mel1700 cells reveals diffuse nuclear staining as a marker of spontaneous or drug-induced lytic replication. Infected MeWo-KSHV (a) and Mel1700-KSHV cells (b) were plated in chamber slides and allowed to adhere overnight, then fixed, permeabilized, and stained with the LANA-specific LN35 primary antibody, followed by chicken anti-rat secondary antibody conjugated to Alexa-Fluor-647 (Cy5). Shown are 63x magnification (oil immersion) images of infected MeWo-KSHV (a) and Mel1700-KSHV cells (b) showing LANA (top image) along with the corresponding DAPI nuclear counter-stain (bottom image), respectively. Note the predominantly discrete “punctate” LANA staining in MeWo-KSHV cells (a) in contrast to the more diffuse LANA staining in almost all Mel1700-KSHV cells (b).

KSHV LANA maintains viral latency in part by tethering episomal DNA to the host chromosome and by suppressing RTA-controlled lytic genes [49]. Consistent with this function, LANA is often detected as punctate nuclear speckles depicting discrete foci of LANA-mediated tethering of viral episomes to host DNA [49]. In light of our finding that RTA is robustly expressed in Mel1700-KSHV cells even in the absence of drug induction, we speculated that deregulated expression of LANA might relieve RTA repression, resulting in the relatively higher level of virus reactivation in Mel1700, but not in MeWo cells. Consistent with this prediction, all infected MeWo-KSHV cells exhibited punctate nuclear LANA staining that is also typically seen in latently-infected endothelial cells and PEL-derived cell lines [49], whereas LANA staining was predominantly “diffuse” in Mel1700-KSHV cells (Figure 4 and supplementary Figure S5). The “punctate” versus “diffuse” distinction was not due to antibody cross-reactivity or artifacts associated with the IFA, because similar results were obtained in a parallel experiment in which we used a goat anti-rat secondary IgG conjugated to a different fluorophore (Figure S6). Moreover, no background fluorescence was seen in control experiments in which only primary or secondary antibody was used (Figure S7), and, in this case the RFP signal is a result of NaB treatment, which induces a higher level of RFP expression in Mel1700-KSHV cells compared to MeWo-KSHV cells (as illustrated in Figure 2).

To confirm whether diffuse LANA staining directly correlates with lytic replication, we treated both MeWo-KSHV and Mel1700-KSHV cells with NaB and then attempted to simultaneously capture both punctate (unreactivated) and diffuse (reactivated) LANA images in the same cell population. Figure S8 is a representative set of RFP, GFP, DAPI, and LANA images from two separate visual fields I and II (panel A, for MeWo-KSHV) and III and IV (panel B, for Mel1700-KSHV). In MeWo-KSHV cells, diffuse LANA staining was detected only in reactivated (RFP+) cells nos.10, 11, and 12 that are surrounded by apparently nonreactivated (RFP−) cells nos.1–9 in which LANA is indeed punctate (Figure S8A). On the other hand, cells nos.1–6 in the Mel1700-KSHV fields appear to be reactivated, and accordingly exhibit diffuse LANA staining (Figure S8B), confirming that diffuse nuclear LANA staining may be a marker for lytic replication, while punctate LANA expression may reflect viral genomes in a predominantly latent state. Surprisingly, we also detected cytoplasmic LANA staining in a small number of reactivated cells (e.g., MeWo-KSHV cells #10–12) and Mel1700-KSHV cells (#1 and 3). Although the molecular basis for extranuclear LANA staining in reactivated cells is not known, it reveals important new perspectives on hitherto unrecognized regulatory functions for novel LANA isoforms that may control the timing, robustness, and molecular threshold for the mechanisms that control viral reactivation.

### 3.5. Differential Processing and Expression of KSHV LANA Reveal Novel Mechanisms for Regulation of the Viral Lytic Cycle in Skin-Derived Cells

**Figure 5:**
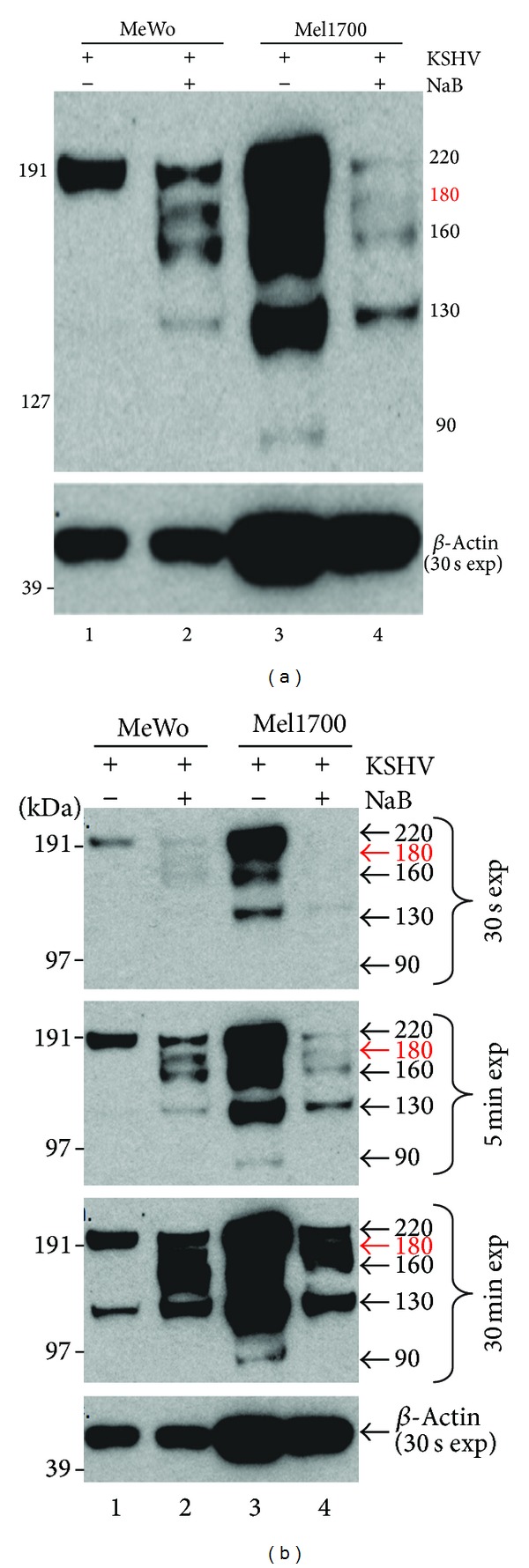
Differential expression of specific LANA isoforms in KSHV-infected MeWo and Mel1700 cells is associated with differences in viral lytic replication. (a) RIPA buffer lysates of rKSHV.219-infected MeWo and Mel1700 cells either untreated (−) or treated with NaB (+) for four days were resolved in SDS-PAGE and probed with anti-LANA antibody LN35. Blots were visualized with anti-rat-horse radish peroxidase (HRP)-conjugated secondary antibody. (b) 30 sec, 5 min., and 30 min. exposure times for the blot in (a), revealing “fine” differences between the samples with respect to expression of specific LANA bands. *β*-Actin (30 sec. exposure) was used as loading control, and the estimated LANA band sizes are on the right side of the blot(s).

Since the LANA genome-tethering function depends on its ability to bind host DNA, and since entry into the viral lytic cycle may disrupt these interactions in a manner that could, in theory, result in delocalized (diffuse) LANA staining, we tested the hypothesis that diffuse LANA staining during lytic replication reflects a disproportionate accumulation of C-terminally truncated LANA isoforms that lack the capacity to bind DNA and are, therefore, diffusely expressed throughout the nucleus and possibly the cytoplasm as well. Interestingly, KSHV LANA (1,162 aa) has a predicted molecular weight of 135 kDa, yet various studies have often detected the protein as a doublet of approximately 220–230 kDa in addition to two NaB-inducible 150–180 kDa and 130 kDa bands [49], underscoring the potential for multiple posttranscriptional regulation. In the current study, we also detected the 220 kDa band in uninduced MeWo-KSHV lysates (Figure 5(a), lane 1), in addition to a 130 kDa band that was induced by NaB (Figure 5(a), lane 2) but became clearly visible in uninduced MeWo-KSHV samples only after a 30 min. exposure (Figure 5(b), lane 1). Mel1700-KSHV cells also contained the ~220 and 130 kDa bands in addition to three more bands at ~180, 160, and 90 kDa (Figure 5(a), lane 3), all of which were expressed in absence of NaB induction. Although the *β*-actin control band suggests that less total protein may have been loaded for the MeWo samples, additional repeats and longer exposures confirmed that the MeWo samples expressed the ~180, 160, or 90 kDa bands only after NaB treatment (Figure 5(b), compare lanes 1 and 2), in contrast to Mel1700-KSHV cells in which they were clearly present even in absence of NaB induction, giving us reason to believe that the ~180, 160, and 90 kDa LANA bands may be uniquely associated with lytic replication. We note that the reduction in LANA-specific bands in NaB-treated Mel1700-KSHV cells (Figure 5(a), lane 4) may be either due to NaB cytotoxicity or cell lysis potentially associated with infectious virus release. Another insightful finding was that the pattern of LANA expression in infected MeWo cells was analogous to BCBL-1 cells in which LANA is often detected as punctate speckles [49], a fact that is even more demonstrable in the side-by-side western blots (Figure S9A) and LANA IFA images (Figure S9B). Together, these results imply that punctate LANA staining marks strong latency, whereas the switch to diffuse staining may represent entry into the viral lytic phase.

### 3.6. NF-*κ*B Activation and the Overall Inflammatory Status of Infected Cells Regulate KSHV Latency in Skin Cells

**Figure 6:**
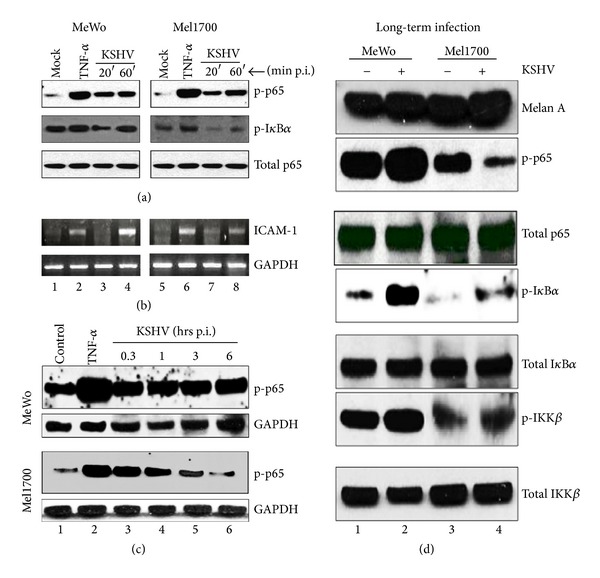
Early activation of NF-*κ*B signaling during acute infection with rKSHV.219 is sustained in MeWo cells, but subsequently limited in Mel1700 cells following long-term infection. (a) MeWo and Mel1700 cells either mock-infected, treated for 20 min. with TNF-*α*, or infected with rKSHV.219 for 20 and 60 min. were used in western blot analysis for phosphorylated NF-*κ*B p65. Mock-infected cell lysates were used to determine baseline levels of phosphorylated NF-*κ*B p65, whereas lysates from cells treated with TNF-*α* served as positive controls for activation of NF-*κ*B p65. Total NF-*κ*B protein was used as an internal loading control. (b) Total RNA from half of the same cells used in (a) was used for RT-PCR amplification of the NF-*κ*B-controlled ICAM-1 gene. (c) MeWo cells (top) and Mel1700 cells (bottom) either mock-infected (control), TNF-*α*-treated, or infected with rKSHV.219 for 0.3 h, 1 h, 3 h, or 6 h were used in western blot analysis for phosphorylated NF-*κ*B p65. (d) Western blot analysis of the phosphorylation levels of key components of the NF-*κ*B-signaling pathway in long-term cultures of uninfected (−) and infected (+) MeWo and Mel1700 cells.

A number of studies have shown that a preexisting state of inflammation or direct KSHV-mediated activation of the NF-*κ*B pathway early following infection may be necessary for successful establishment of viral latency [50, 51]. To examine this concept in skin cells, we analyzed the activation status of key components of the classical NF-*κ*B pathway in relation to intrinsic cellular attributes that support specific virologic outcomes during both *de novo* (or acute) and persistent (or chronic) infection. Similar to previous reports, KSHV rapidly induced phosphorylation of NF-*κ*B p65 and IkB*α* within 20 minutes of infection (Figures 6(a) and 6(b)), an effect that correlated with changes in expression of the NF-*κ*B-controlled intracellular adhesion molecule 1 (ICAM-1) [52]. Interestingly, while phosphorylated p65 remained relatively unchanged for several hours postinfection of MeWos, it was short-lived and in fact began to decline within one hour postinfection of Mel1700 cells (Figure 6(c)). Since NF-*κ*B activation represses RTA while its inhibition relieves RTA repression [17, 18], the fact that NF-*κ*B activation is curtailed in KSHV-infected Mel1700 cells implies that Mel1700 cells likely express endogenous mechanisms that function in an adaptive manner to attenuate NF-*κ*B activity, a process that could explain the relatively higher propensity for virus reactivation in these cells. Consistent with this view, we found that after several days of selective passage in presence of puromycin (designed to maintain stable replicative viral genomes in persistently infected cells), key components of the NF-*κ*B signaling axis were differentially impacted by the virus, even though both MeWo and Mel1700 cells retained their melanoma phenotype, as indicated by sustained expression of Melan-A (Figure 6(d)). For example, while phosphorylated p65, IkB*α*, and IKK*β* were elevated in persistently infected MeWos, the level of phosphorylated p65 was dramatically reduced, and the relative increase in phosphorylated IkB*α* and IKK*β* was also much more modest in long-term cultures of infected Mel1700 cells (Figure 6(d), compare lanes 2 and 4, resp.). Therefore, we have found that, in comparison to MeWo cells, Mel1700 cells (a) support a more robust viral lytic replication program (Figure 2), (b) express higher levels of RTA (Figure 3), (c) display diffuse nuclear LANA staining (Figure 4(b)) that correlates with stage-specific expression of specific LANA isoforms including the 180 kDa band that is associated with lytic replication (Figure 5), and (e) have reduced NF-*κ*B activation (Figure 6). These dichotomous properties are consistent with a model whereby sustained activation of NF-*κ*B (as in MeWo cells) correlates with maintenance of latency whereas a reduction in activated NF-*κ*B p65 (as in Mel1700) correlates with a higher propensity for viral reactivation, a paradigm supported by findings in other cell types as well [17, 18, 50, 51].

### 3.7. KSHV Blocks Expression of Anti-Inflammatory Response Proteins to Maintain a Latent State in MeWo Cells, but Fails to Do so in Mel1700 Cells That Support Robust Lytic Replication

**Figure 7:**
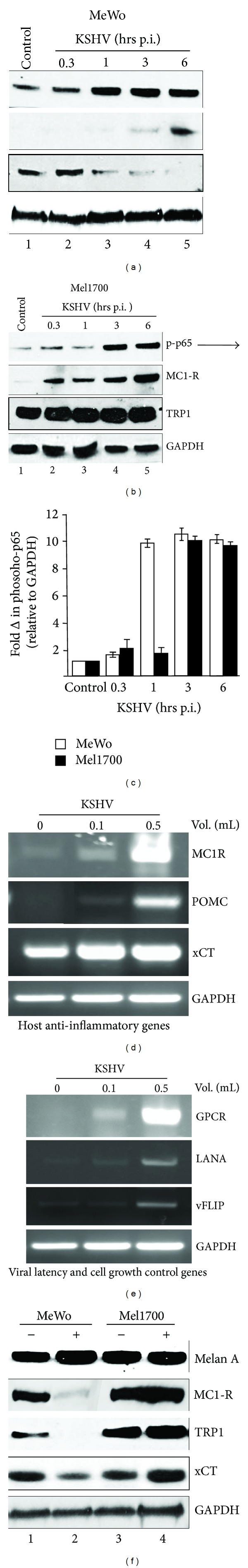
A link between KSHV latency and the MC1-R signaling axis in skin-derived cell lines. (a) Western blot analysis of phosphorylated NF-*κ*B p65, MC1-R, and TRP-1 in total cell lysates extracted from MeWo (a) or Mel1700 (b) cells either uninfected (control) or acutely infected with KSHV for 0.3 h, 1 h, 3 h, or 6 h. GAPDH was used as loading control. (c) ImageJ quantitation of the p65 band intensities in (a) and (b) relative to GAPDH controls. (d) Mel1700 cells were infected in 6-well plates with increasing volumes (mL/well) of concentrated supernatant containing infectious KSHV, and total RNA from infected cells was subjected to RT-PCR using primer sets for host anti-inflammatory MC1R, POMC, and SLC7A11. (e) Equal aliquots from the same RNA used in (d) were subjected to RT-PCR analysis for select viral latency and cell growth control genes (i.e., GPCR, LANA, and v-FLIP). (f) Western blot analysis of the melanoma cell marker, Melan A, and anti-inflammatory genes MC1-R, TRP1, and SLC7A11 in total cell lysates of uninfected (−) or chronically infected (+) long-term cultures of MeWo-KSHV and Mel1700-KSHV cells. GAPDH was used as an internal control for both the RT-PCR (e) and western blot assays.

The control of inflammation within the epidermal unit of skin is orchestrated by anti-inflammatory responses primarily initiated by alpha-melanocortin stimulating hormone (*α*-MSH), a potent peptide derived from pro-opiomelanocortin (POMC) by posttranslational proteolytic processing [53]. The effects of *α*-MSH are transduced through the melanocortin 1 receptor (MC1-R) [54], which is expressed by skin-resident melanocytes, keratinocytes, microvascular endothelial cells, and other monocytic infiltrates [54]. Upon binding to MC1-R, *α*-MSH triggers a signaling cascade that effectively blunts inflammatory reactivity by preventing activation of NF-*κ*B and the associated expression of pro-inflammatory cytokines and adhesion molecules that mediate infiltration of pro-inflammatory cells into the vascular endothelial environment [53, 55–57]. *α*-MSH also regulates pigment production and deposition through activation of tyrosinase, the enzyme that catalyzes synthesis of dopaquinone, the first step in melanogenesis [58]; in turn, dopaquinone reacts with intracellular cystine (supplied by the cystine/glutamate transporter, xCT) to produce cysteinyl-dopa, a rate-limiting step in the synthesis of pheomelanin during inflammation [59]. Since establishment of a persistent state is determined by the overall inflammatory status of infected cells, we asked whether establishment of strong KSHV latency (in MeWo and not in Mel1700) is controlled by differential deregulation of endogenous anti-inflammatory mediators that would otherwise be expressed to prevent establishment of latency in these cells. We measured the temporal changes in NF-*κ*B activation in relation to key components of the anti-inflammatory *α*-MSH/MC1-R signaling axis, and found that within the first 1 hr of infection KSHV induced a rapid increase in phosphorylated NF-*κ*B p65 in MeWo cells (Figure 7(a)). This was in contrast to Mel1700 cells in which the increase in NF-*κ*B activation did not occur until at least 3 hrs postinfection (see Figure 7(b) and the ImageJ quantitation of phosphorylated p65 band intensities in Figure 7(c)). During the rapid increase in NF-*κ*B activation in MeWos cells, MC1-R was not expressed while there was a progressive abrogation of endogenous tyrosinase-related protein-1 (TRP-1), which is involved in pigment synthesis and deposition during inflammation (Figure 7(a)). By contrast, MC1-R protein was induced almost immediately after infection of Mel1700 cells, but there was no appreciable impact on the already high baseline level of TRP-1 (Figure 7(b)); instead, there was a virus-induced increase in mRNA for MC1-R, POMC (the precursor of *α*-MSH), and xCT (gene name SLC7A11) (Figure 7(d)). This effect correlated with the amount of virus used, as marked by the correspondingly dose-dependent expression of viral GPCR, LANA, and vFLIP (Figure 7(e)).

When infection was monitored over a longer period of time, it was evident that phosphorylated p65 remained high in MeWos, whereas in Mel1700 cells it was consistently detected much later (Figure S10A). Moreover, the kinetics of phosphorylated p65 in both cell lines overlapped with expression of xCT (Figure S10B), which not only forms a receptor complex that mediates KSHV entry [39], but also supplies cystine for the manufacture of glutathione (GSH), a potent anti-inflammatory mediator [60]. Interestingly, we recently discovered that KSHV microRNAs target a transcriptional inhibitor of xCT in infected cells, ostensibly to promote virus dissemination while protecting infected cells from inflammatory stress [61]. Therefore, the fact that the kinetics of NF-*κ*B activation overlap with xCT expression in KSHV-infected skin cells is consistent with cellular induction of anti-inflammatory responses as an adaptive response to inflammation during the latent state. In accordance with this link, we detected a significant reduction in the expression levels of xCT, MC1-R, and TRP-1 in persistently infected MeWos, but in Mel1700 cells expression of MC1-R and TRP-1 was sustained in conjuction with an increase in xCT (Figure 7(f)). Together, these findings suggest that the differential influences of KSHV in MeWo and Mel1700 that we observed following *de novo* infection are also maintained after establishment of latency.

### 3.8. KSHV Latency Correlates with Impairment of the Overall Anti-Inflammatory Response in Infected Skin Cells

**Figure 8:**
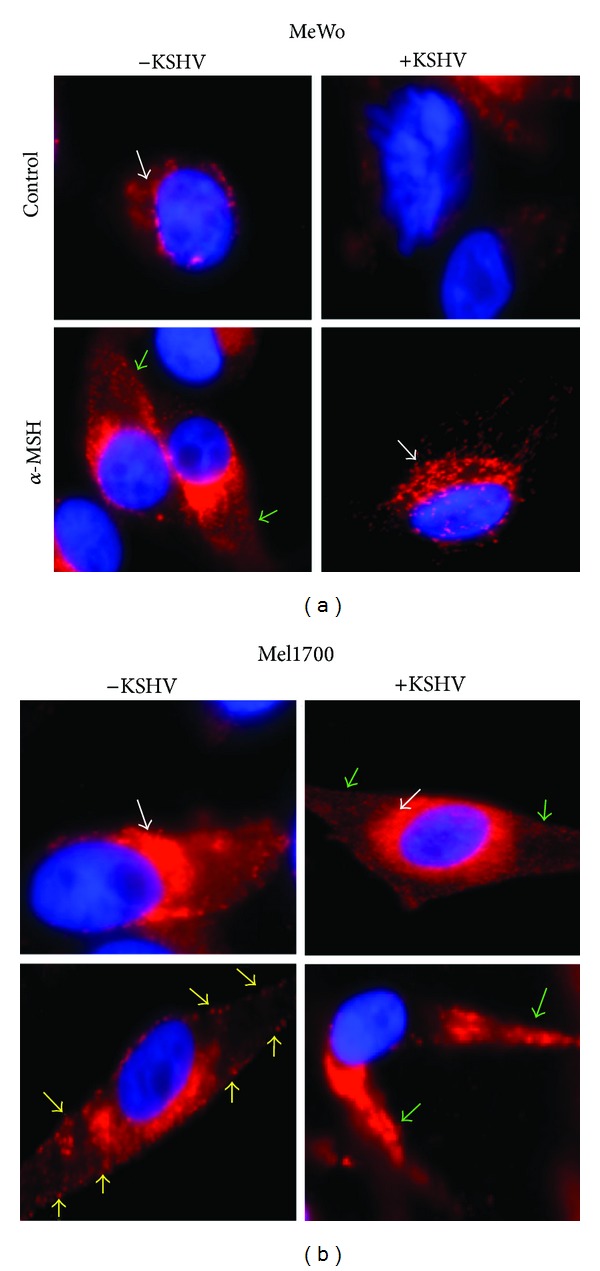
KSHV impairment of the melanogenic response is a virologic correlate of latency in skin cells. Uninfected (−) and rKSHV.219-infected (+) MeWo cells (a) or Mel1700-cells (b) were plated on chamber slides without (control) or with *α*-MSH and allowed to adhere overnight. Cells were then fixed, permeabilized, and stained with anti-TRP-1 antibody and counterstained with DAPI nuclear stain. Shown are representative 63x magnification (oil immersion) images of representative cells from each field of view. White arrows depict ER/Golgi-associated TRP-1 (apparently present in untreated MeWo and in *α*-MSH-treated MeWo-KSHV cells), whereas green arrows point to outgrowths of promelanogenic dendritic spines induced by *α*-MSH in MeWos ((a) bottom left) and in KSHV-infected Mel1700, in which they appear to increase even more profoundly upon treatment with *α*-MSH (b). For wide-view 20x images of the same treatments, please see Figure  S11.

To determine the biological significance of the impact of KSHV on mediators of the endogenous melanogenic response, we treated both uninfected and KSHV-infected MeWo or Mel1700 with *α*-MSH and assessed the cellular redistribution of TRP-1, a known downstream marker for the effector function of MC1-R activity. TRP-1 is expressed exclusively in melanosomes where it plays a role in the maintenance of melanosome structure. As shown in Figure 8, untreated MeWo cells express weak but detectable levels of TRP-1 that is predominantly associated with the ER/Golgi network (Figure 8(a), top-left quadrant). However, treatment with *α*-MSH resulted in a slight change in the distribution of the TRP-1 from being exclusively associated with the ER/Golgi to other aspects of the cell (Figure 8(a), lower-left images), indicating that these cells are capable of responding to the anti-inflammatory effects of *α*-MSH. However, in KSHV-infected MeWos, the basal expression of TRP-1 was abrogated (Figure 8(a), top-right image), consistent with western blot data in Figure 7(a). Remarkably, treatment of KSHV-infected MeWo-KSHV cells with *α*-MSH restored TRP-1 expression to levels analogous to those in uninfected samples (Figure 8(a), white arrows), revealing a direct influence of KSHV on the MC1-R/*α*-MSH axis in these cells. On the other hand, we found that unlike MeWo and their infected counterparts in which not all cells expressed TRP-1, Mel1700 cells expressed high basal levels of TRP-1 (Figure 8(b), top-left image), and in this case KSHV actually did not abrogate but instead induced a noticeable increase in TRP-1 expression (Figure 8(b), top-right image; also see TRP-1 blot in Figure 7(b)). Moreover, *α*-MSH treatment of Mel1700-KSHV cells resulted in a dramatic alteration in TRP-1 expression from being primarily associated with the supranuclear ER/Golgi network to what appeared, at higher magnification, to resemble melanogenic vesicles arrayed along dendritic spines (Figures 8(b) and S11B, green arrows, and additional data not shown). At a functional level, it is reasonable to conclude that in MeWos that fail to express the necessary anti-inflammatory mediators in the context of infection, viral latency is the more likely outcome, whereas the rapid increase in expression of MC1-R and other anti-inflammatory molecules in infected Mel1700 cells blocks NF-*κ*B [53, 55–57], which would consequently limit establishment of a latent state and/or result in a greater propensity for Mel1700 cells to support lytic replication.

## 4. Discussion

In this study, we investigated the infectious process of KSHV in skin-derived cells in order to identify pathogenetic themes that underlie the ability of KSHV to induce cutaneous KS lesions in skin, a highly specialized site whose endogenous anti-inflammatory processes presumably fail to overcome this pathologic outcome. Using primary melanocytes as well as established melanoma-derived cells, we examined key markers of virus/host interactions that control KSHV tropism and pathogenesis in skin and identified important correlates that link the replicative program of KSHV with the underlying inflammatory status of infected cells. Specifically, we identified two cell lines—MeWo and Mel1700—that consistently displayed differences in (a) susceptibility to productive infection, (b) timing and robustness of virus replication, (c) expression of KSHV latency-associated nuclear antigen, LANA, (d) replication phase-specific expression of viral genes, and (e) susceptibility to virus-induced perturbation of the anti-inflammatory melanogenic response. We found that underlying these differences are cell-type specific markers that operate at the level of KSHV interactions with the host genome, chief among them being differential expression of KSHV LANA.

LANA maintains viral latency in part by tethering episomal viral DNA to host chromosomes and by blocking RTA expression [49]. We found that diffuse nuclear LANA staining was associated with lytic replication, whereas punctate staining marked a state of latency, leading us to hypothesize that diffuse LANA staining reflects the accumulation of “untethered” LANA isoforms that are unable to block RTA. Consistent with this view, NaB induced a switch from punctate to diffuse LANA staining; moreover, RTA expression was higher in spontaneously reactivated Mel1700-KSHV cells in which LANA staining was diffuse, in contrast to MeWo-KSHV cells in which RTA was expressed only after NaB treatment, further supporting our hypothesis.

Although the mechanisms that control nuclear LANA expression at different stages of the virus life cycle are not fully known, it was recently observed that when the N- and C-termini of LANA (that contain nuclear localization signals, NLS) were expressed individually in KSHV-infected cells, the C-terminus (which binds terminal repeats of KSHV DNA) formed discrete nuclear speckles, whereas the N-terminus (which binds host chromosomes) exhibited diffuse nuclear staining [62–64]. However, when full length LANA was expressed by itself in uninfected cells, it exhibited diffuse nuclear staining that became speckled when a bacterial artificial chromosome containing the KSHV terminal-repeat region was introduced [65]. Furthermore, a naturally occurring LANA isoform (with a 76 amino acid truncation at the C-terminal end) was detected in KSHV-infected primary effusion lymphoma (PEL) cell lines, BCP-1 and BC-3, but this isoform was incapable of binding to KSHV episomes and therefore exhibited diffuse nuclear staining [66]. Together, these findings support the conclusion that diffuse LANA staining reflects the accumulation of LANA isoforms that may still be associated with host chromatin but are not tethered to viral genomes presumably undergoing a state of lytic replication. Surprisingly, we also detected diffuse LANA staining in the cytoplasm of a few reactivated Mel1700-KSHV cells, and although there is currently no published evidence for nucleo-cytoplasmic shuttling of functional LANA in KSHV-infected cells, one study showed that the LANA amino acids 1–323 region, which lacks a NLS, is retained in the cytoplasm [67]. Whether this short form of LANA is generated and shuttled to the cytoplasm in some lytically replicating cells remains to be determined.

Various studies have consistently shown that depending on the cell type and the state of viral reactivation, the LANA-specific LN35 antibody used in this study can detect at least five distinct LANA bands at approximately 220, 180, 160, 130, and 90 kDa, that are potentially generated by alternative initiation [68], truncations [66], or post-translational modifications [69–71]. Because the epitope recognized by LN35 has not yet been mapped, the identity and amino-acid sequence(s) of each of these bands requires systematic proteomic analysis beyond the scope of the current study. Nonetheless, we and others have now shown that appearance of the ~180, 130, and 90 kDa LANA-specific bands correlates with lytic replication [72, 73], which leads us to believe that the ~220 kDa band likely corresponds to full-length LANA, while the ~180 kDa band may be the C-terminally truncated form associated with diffuse LANA staining [66] because it is present only in lytically replicating cells. One prediction from these findings is that the ratio of the 180 kDa band relative to full length LANA regulates the switch from the latent to the lytic phase. Interestingly, Toptan et al. recently described complex LANA isoforms analogous to those that we have detected and proposed that these bands may result from noncanonical translation initiation [68]. While these independent findings reveal an emerging new perspective on the complexity of LANA function(s), they also raise several new questions. (a) What factors trigger non-canonical translation initiation and/or differential processing of LANA into products that may be shuttled to the cytoplasm? (b) Do cells such as Mel1700 that display a higher propensity for supporting lytic replication express host restriction factors that induce the generation of cytoplasmic LANA isoforms that are unable to mediate host chromosomal tethering to the virus genome? (c) What regulatory mechanisms control the accumulation of specific LANA isoforms in relation to full length LANA? Clearly, the pathogenetic implications of these regulatory themes cannot be over-emphasized, but they are not unprecedented among the gammaherpesviruses. For example, during infection with Epstein-Barr virus (EBV), a gammaherpesvirus closely related to KSHV, at least eight different transcripts of the EBV latency-associated nuclear antigen (i.e., EBNA-1, 2, 3A, 3B, 3C, 4, 5, 6), are expressed to control various states of the EBV latency program [74]. Since EBNA is the functional homolog of KSHV LANA, it is conceivable that LANA analogs of the various EBNA isoforms may also be expressed at specific stages of the KSHV life cycle, although further investigation will be required in order to isolate replication phase-specific functions of the protein in a given cellular context.

Our study also revealed a direct link between the NF-*κ*B activation status and virus reactivation, consistent with previous findings that NF-*κ*B activation early following KSHV infection is required for establishment and maintenance of latency [17, 18] and for oncogenic transformation [19, 20]. Interestingly, the KSHV latency program in MeWo cells was accompanied by sustained activation of NF-*κ*B, coincident with reduced expression of key markers of the endogenous anti-inflammatory response. Conversely, in Mel1700 in which the virus readily undergoes spontaneous lytic replication, phosphorylated NF-*κ*B p65 remained low, concomitant with sustained expression of anti-inflammatory proteins, leading us to conclude that KSHV may persist in skin cells both by regulating its replicative program through activation of NF-*κ*B, while also exerting virus-induced subversion of the anti-inflammatory axis. Therefore, it would be reasonable to speculate that in order for KSHV to persist and cause cutaneous disease in skin, it must infect and then either directly or paracrinally overcome the endogenous anti-inflammatory reactions of skin-resident cells in favor of conditions that support development of KS. Indeed, we found that KSHV can indeed infect and induce cytologic reprogramming in melanocytes, the key cellular sensors of inflammatory injury in skin.

**Figure 9:**
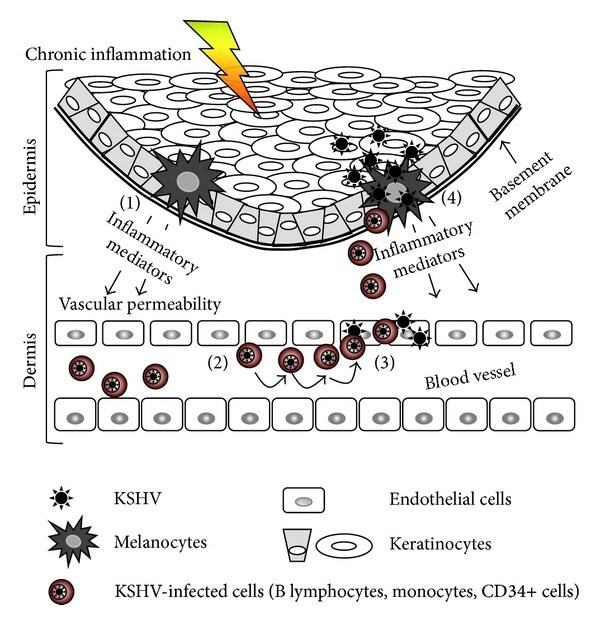
Hypothetical model of immunophysiologic mechanisms that control transmission of KSHV from latently infected cellular reservoirs to cells within skin. (1) Chronic activation of NF-*κ*B in the skin induces expression of adhesion molecules on the surfaces of endothelial cells, increased vascular permeability, and secretion of inflammatory cytokines and chemokines by melanocytes and other resident cells of the skin, (2) recruitment of immune cells and other KSHV-infected monocytes to the vascular endothelium, and (3) extravasation of infected cells from the blood vessels into the underlying dermis, where virus may be transmitted to vascular endothelial cells through cell-to-cell contact or other mechanisms. (4) Accumulation of cell-free and KSHV-infected cells at the superficial dermis, where they interact with and infect resident melanocytes and keratinocytes in the basal membrane.

Prior to this study, melanocytes were not known to be targets for KSHV infection, which might have led some to question their relevance during KSHV pathogenesis. However, it can be argued that the anatomical location and function of melanocytes as sentinels of inflammation at the interface of microbial offense and host immunity [58, 75] support the expectation that these cells might regulate KSHV pathogenesis in skin. In response to infections and other pro-inflammatory cues, melanocytes express a wide range of signaling peptides including *α*-MSH. Upon binding to MC1-R, *α*-MSH transduces signals that prevent activation of NF-*κ*B and the associated expression of pro-inflammatory cytokines, tumor growth factors, and adhesion molecules that mediate transmigration of inflammatory cells through the vessel wall during angiogenic neovascularization [56, 58]. Given the fundamental role of inflammation in KSHV pathogenesis, and the fact that *α*-MSH antagonizes NF-*κ*B [56, 58], the ability of KSHV to infect melanocytes may have profound implications for KS development in skin. If impairment of the expression of anti-inflammatory molecules (such as MC1-R, TRP-1, and xCT) represents an important virologic correlate for establishment of KSHV latency in skin cells, it is likely that settings in which sustained inflammation cannot be overcome would support virus-associated disease, whereas approaches that enhance the anti-inflammatory response (e.g., via *α*-MSH-dependent disruption of NF-*κ*B activation [76]) may disfavor viral persistence in skin and should, therefore, be explored as a potential new option for clinical management of cutaneous KS or other virus-induced dermatologic conditions that may depend on chronic inflammation [77]. Specifically, chronic inflammation in skin may trigger increased vascular permeability, upregulated expression of cell-adhesion molecules on vascular endothelium, and chemokine-mediated recruitment of KSHV-infected cells from the underlying blood vessels to the site of inflammation where seeding of KS might occur (Figure 9), and the fact that monocytes, macrophages, and activated dendritic cells make up a large proportion of the inflammatory infiltrate often present in cutaneous KS lesions [6, 7] supports this model. Additionally, our finding of efficient transmissibility of Mel1700-derived virions to keratinocytes *in vitro* underscores the high probability of a pathophysiologic framework for virus dissemination in and out of skin, which is plausible given the close proximity and dynamic communication networks that naturally exist between melanocytes and keratinocytes in the human epidermal unit [75]. Ironically, such a framework could be exploited by the virus since the anti-inflammatory melanogenic processes of melanocytes that are generally induced as an adaptive response to virus infection could be co-opted for virion transfer. This phenomenon, referred to as “melanosome hijacking”, has already been described for varicella-zoster virus, another herpesvirus that displays tropism for human skin [78].

## 5. Conclusions

Inherently persistent viruses such as KSHV must manipulate infected host cells to support mechanisms that favor either long-term infection within the target organ or inducible productive lytic replication that promotes viral dissemination to other permissive aspects of the host. Consequently, understanding mechanisms that control the balance between viral latency and lytic replication is an important goal that should guide development of strategies for limiting the pathogenesis of KS and other virus-associated diseases that emerge during the persistent state. Here, we found that the balance between underlying inflammation and lytic replication is a fundamental correlate of KSHV latency and, by extension, KSHV-associated pathology. Although the specific cellular and molecular determinants that control the intrinsic ability of certain cell types to support KSHV latency are not fully defined, our study represents an important first step in our understanding of the nature and biologic impact of KSHV-mediated impairment of the anti-inflammatory function in skin cells, with implications for pathologic outcomes such as cutaneous KS whose progression relies on inflammatory reactivity. Unfortunately, we cannot fully explain why the cell lines used in this study displayed such profound differences in the outcomes of KSHV infection. One clue may be revealed by the fact that unlike Mel1700, MeWo is a highly-pigmented cell line with a high level of underlying inflammation [37], which is consistent with their ability to support a strong latency program. Alternatively (or in addition), it is possible that mechanisms for virus reactivation such as virus-induced apoptosis [79, 80] may be expressed in Mel1700 cells and not MeWo cells, an interesting concept that should be the focus of follow-on studies beyond the scope of the current study. In addition, isolation and characterization of correlates that control the timing and aggressiveness of KS at a population level may provide predictive power with respect to stratification of individuals at high risk for development of the lesion based on differential exposure to pro-inflammatory cofactors that foment inflammation, including toxins, UV radiation, allergens, trauma, or persistent infections [81]. We note, for example, that in KSHV/HIV coinfected individuals who are generally at a greater risk of developing the most aggressive form of KS, HIV serves as a cofactor for KS not only through establishment of an immunosuppressed environment but also through upregulation of the KSHV receptor, xCT [82], to facilitate KSHV dissemination. Interestingly, HIV replication also requires NF-*κ*B activity [83], while the anti-inflammatory activity of xCT is directly linked to the balance between the NF-*κ*B and *α*-MSH/MC1-R signaling pathways, which this study has revealed to be important for the KSHV life cycle [84]. Therefore, the ability of KSHV to overcome *α*-MSH-mediated inhibition of NF-*κ*B activity in MeWos (in which the virus establishes strong latency), would not only promote HIV replication [85] but could also establish conditions that support cutaneous KS development. Future evaluation of these indicators in high-risk individuals with KS compared to those who are KSHV seropositive but never develop KS may provide insight into which cellular dynamics predispose an individual to cutaneous KS and which host responses to infection could be harnessed to help prevent lesional histogenesis.

## Supplementary Material

supplementary Figures S1 – S11, and the Table are provided to support the main figures presented in the manuscript. The supplementary Figures is only essential for conceptual clarity and amplification of the main figures, whereas the supplementary Table is provided to disclose the nucleotide sequences of the forward and reverse primer sets that were used in this study; these sequences are therefore useful for efforts to reproduce the PCR and/or RT-PCR amplifications of the presented gene products.
