# Supplementary material for: Kaposi's Sarcoma-Associated Herpesvirus Subversion of the Anti-Inflammatory Response in Human Skin Cells Reveals Correlates of Latency and Disease Pathogenesis
Source: J Skin Cancer. 2014 Feb 17;2014:246076. doi: 10.1155/2014/246076 (PMC3951102; doi:10.1155/2014/246076)
Supplement: Supplementary file 1 — supplementary Figures S1 – S11, and the Table are provided to support the main figures presented in the manuscript. The supplementary Figures is only essential for conceptual clarity and amplification of the main figures, whereas the supplementary Table is provided to disclose the nucleotide sequences of the forward and reverse primer sets that were used in this study; these sequences are therefore useful for efforts to reproduce the PCR and/or RT-PCR amplifications of the presented gene products. [file 246076.f1.pptx]

## Slide 1
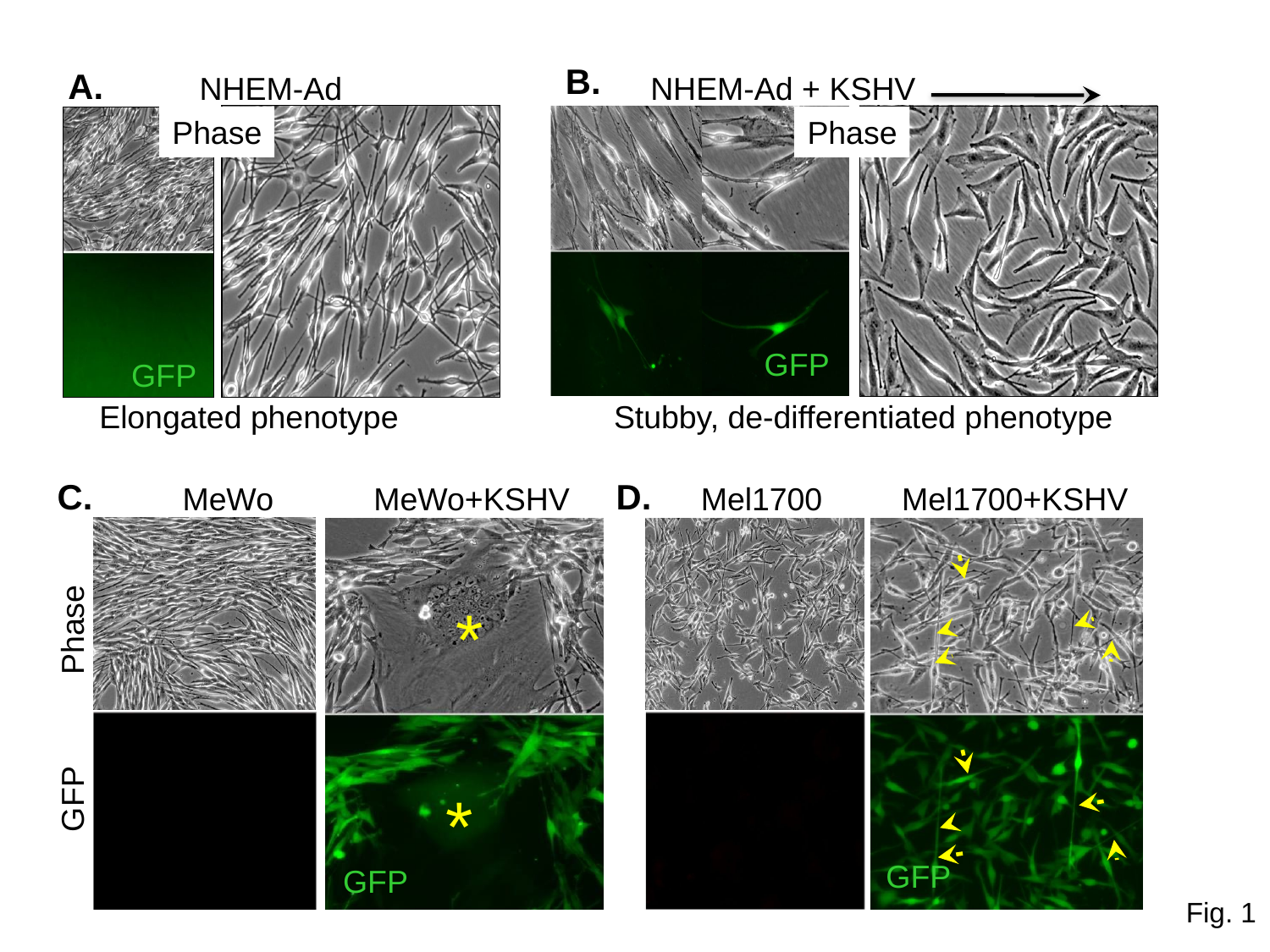

B.
A.
NHEM-Ad
NHEM-Ad + KSHV
Phase
Phase
GFP
GFP
Elongated phenotype
Stubby, de-differentiated phenotype
C.
D.
MeWo
MeWo+KSHV
Mel1700
Mel1700+KSHV
*
*
Phase
GFP
GFP
GFP
Fig. 1

## Slide 2
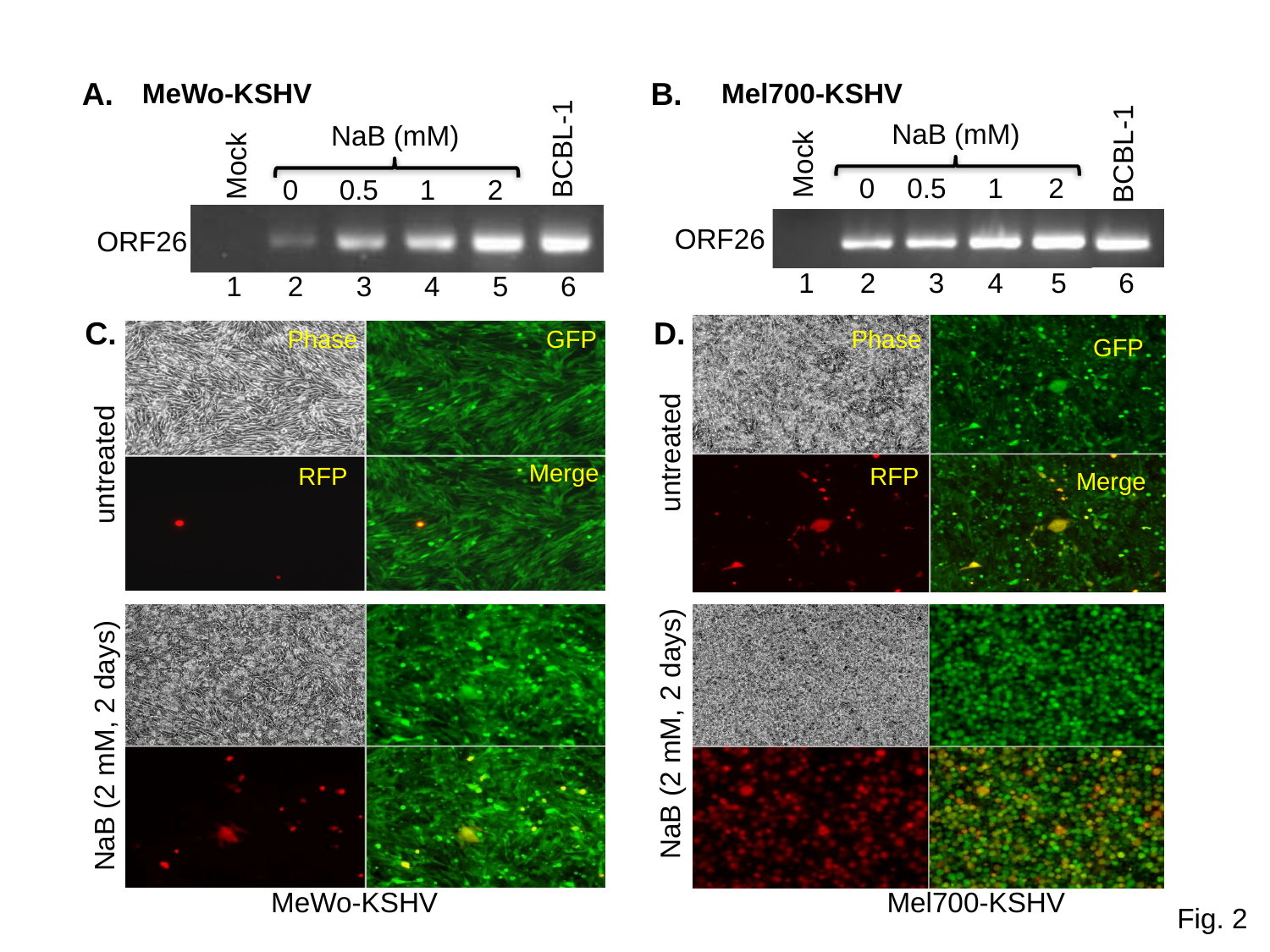

A.
MeWo-KSHV
NaB (mM)
BCBL-1
Mock
0
0.5
1
2
ORF26
B.
Mel700-KSHV
NaB (mM)
BCBL-1
Mock
0
0.5
1
2
ORF26
C.
Phase
GFP
untreated
Merge
RFP
NaB (2 mM, 2 days)
MeWo-KSHV
D.
Phase
GFP
untreated
RFP
Merge
NaB (2 mM, 2 days)
Mel700-KSHV
1
2
3
4
5
6
1
2
3
4
5
6
Fig. 2

## Slide 3
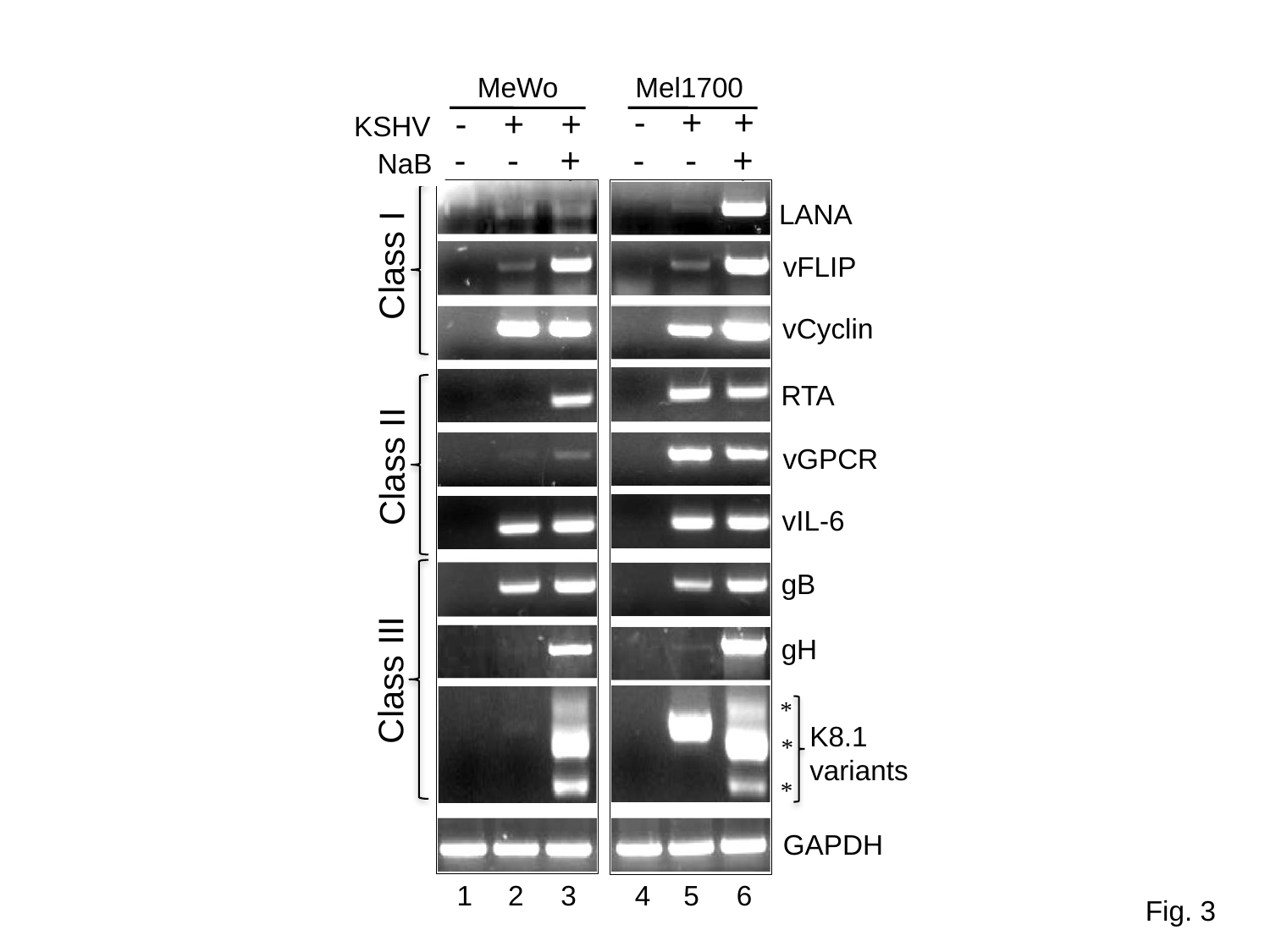

MeWo
Mel1700
-
+
+
-
+
+
KSHV
-
-
+
-
-
+
NaB
LANA
Class I
vFLIP
vCyclin
RTA
Class II
vGPCR
vIL-6
gB
gH
Class III
*
K8.1
variants
*
*
GAPDH
1
2
3
4
5
6
Fig. 3

## Slide 4
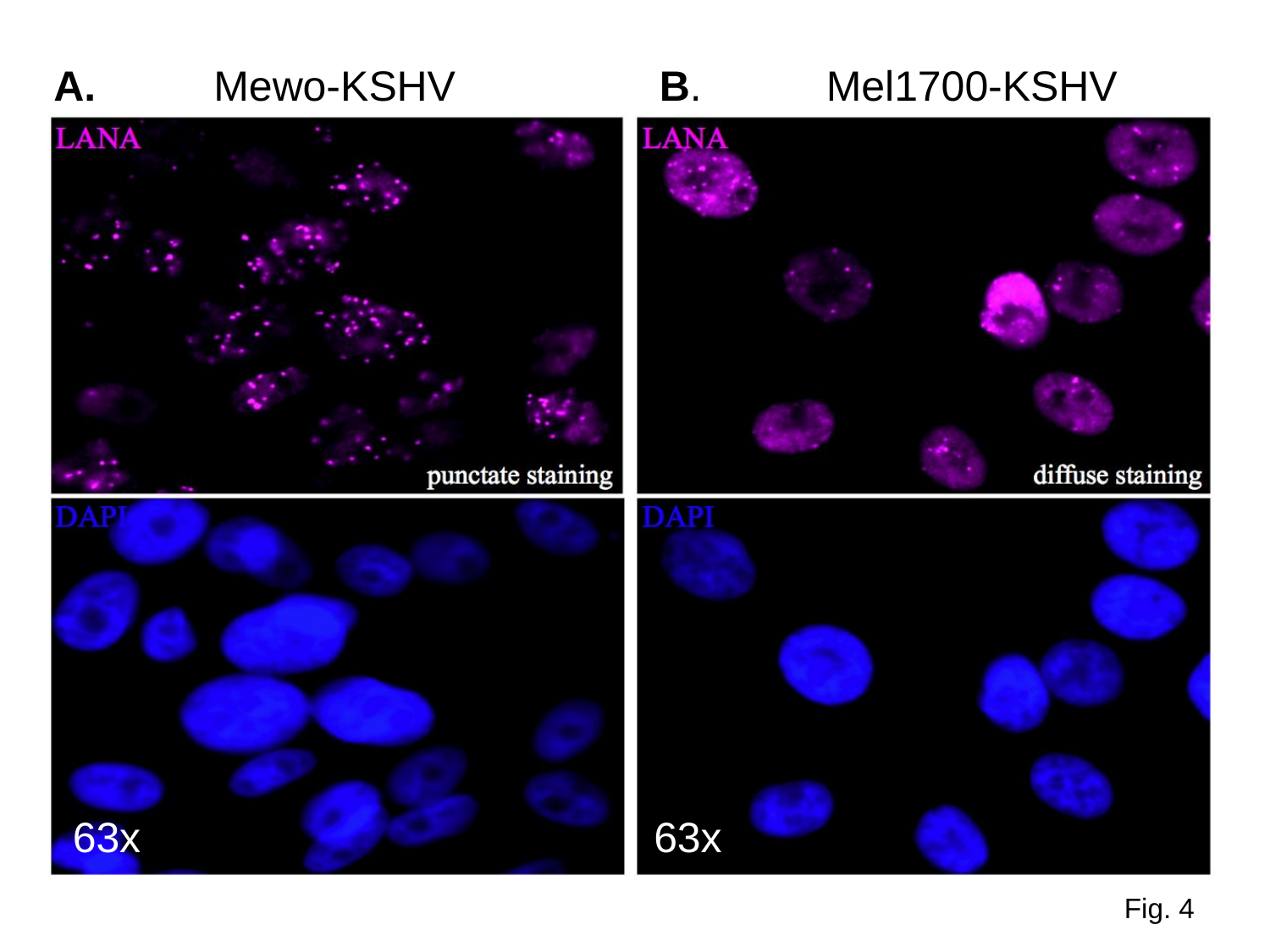

A.
Mewo-KSHV
Mel1700-KSHV
63x
63x
B.
Fig. 4

## Slide 5
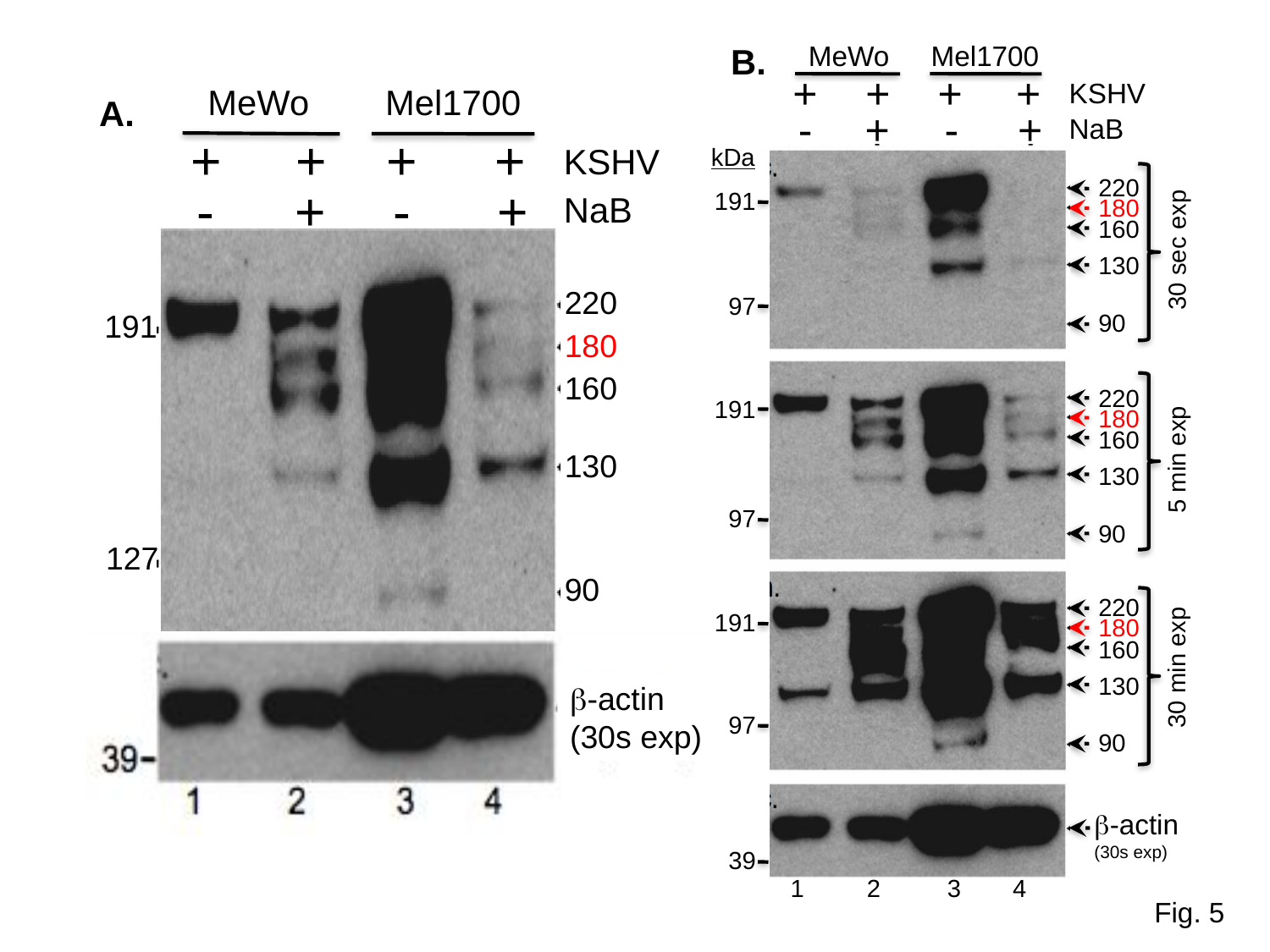

MeWo
Mel1700
B.
+
+
+
+
KSHV
MeWo
Mel1700
A.
-
+
-
+
NaB
+
+
+
+
KSHV
kDa
220
191
180
160
30 sec exp
130
97
90
220
191
180
160
5 min exp
130
97
90
220
191
180
160
30 min exp
130
97
90
b-actin
(30s exp)
39
-
+
-
+
NaB
220
191
180
160
130
127
90
b-actin
(30s exp)
1
2
3
4
Fig. 5

## Slide 6
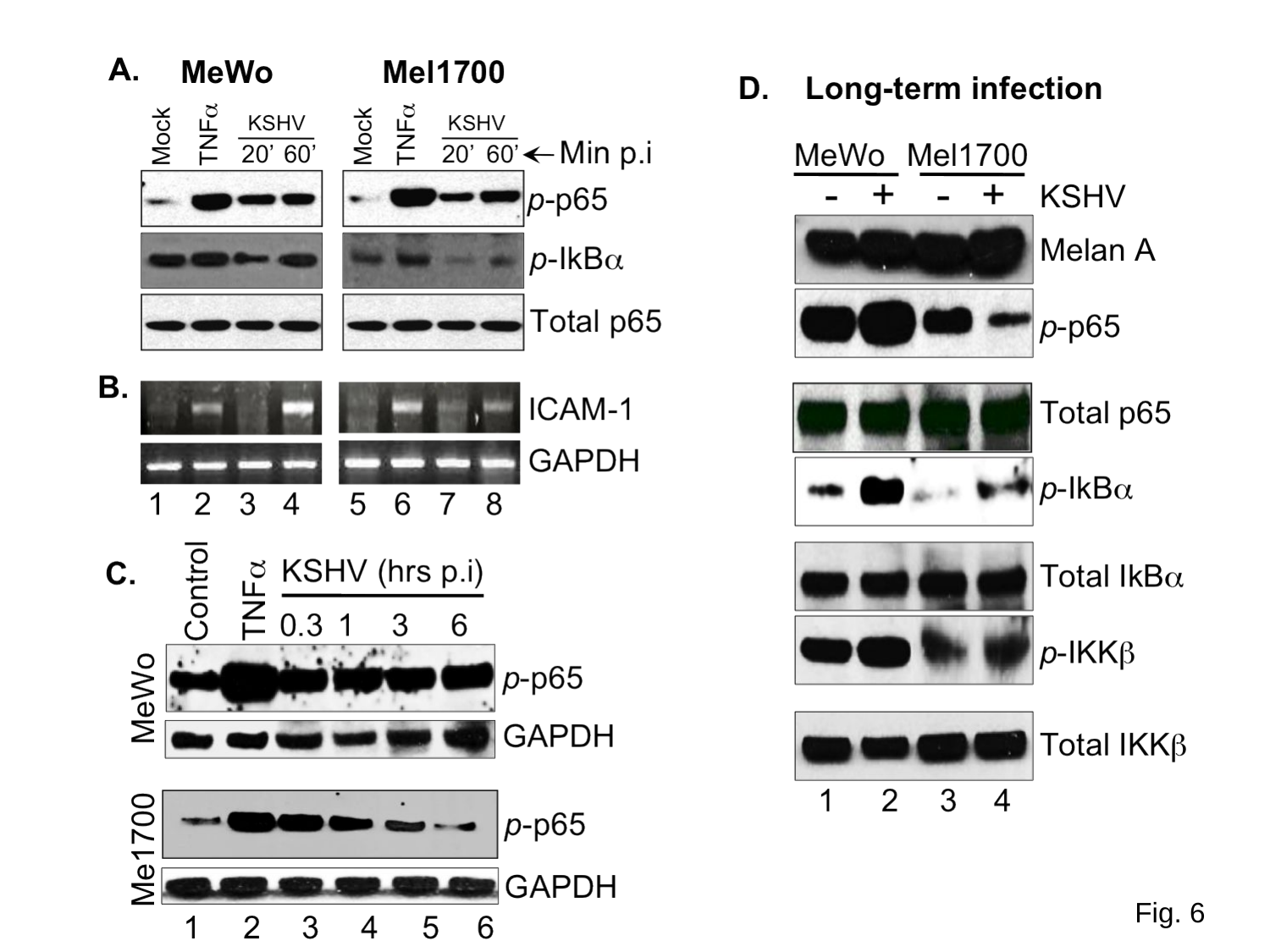

Fig. 6

## Slide 7
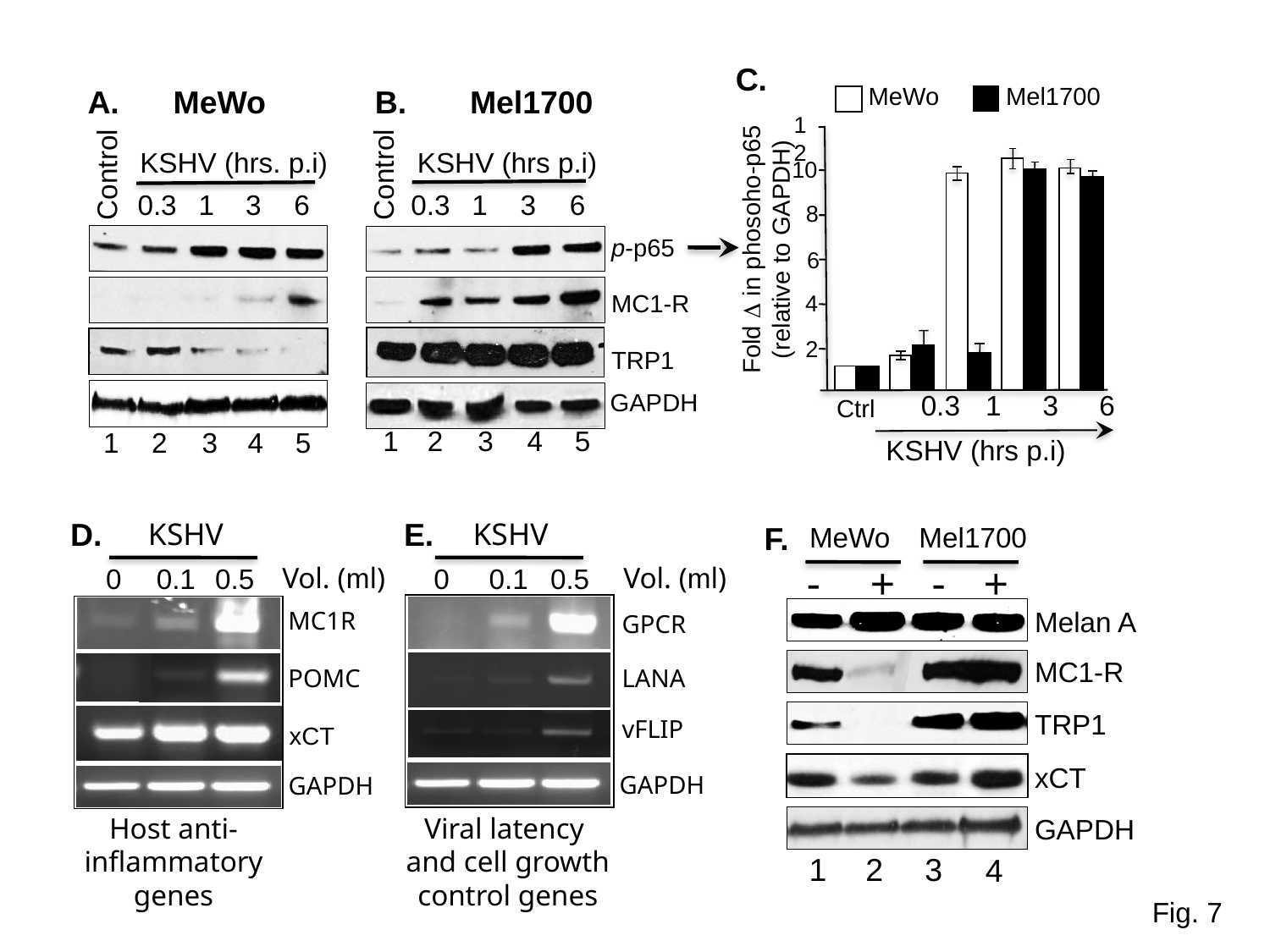

C.
MeWo
Mel1700
12
10
8
6
4
2
Fold D in phosoho-p65
(relative to GAPDH)
Ctrl
KSHV (hrs p.i)
Mel1700
A.
B.
MeWo
KSHV (hrs. p.i)
KSHV (hrs p.i)
Control
Control
0.3
1
3
6
0.3
1
3
6
p-p65
MC1-R
TRP1
GAPDH
1
2
3
4
5
1
2
3
4
5
0.3
1
3
6
D.
E.
KSHV
KSHV
Vol. (ml)
Vol. (ml)
0
0.1
0.5
0
0.1
0.5
MC1R
GPCR
POMC
LANA
vFLIP
xCT
GAPDH
GAPDH
Host anti-inflammatory genes
Viral latency
and cell growth control genes
F.
MeWo
Mel1700
-
+
-
+
Melan A
MC1-R
TRP1
xCT
GAPDH
1
2
3
4
Fig. 7

## Slide 8
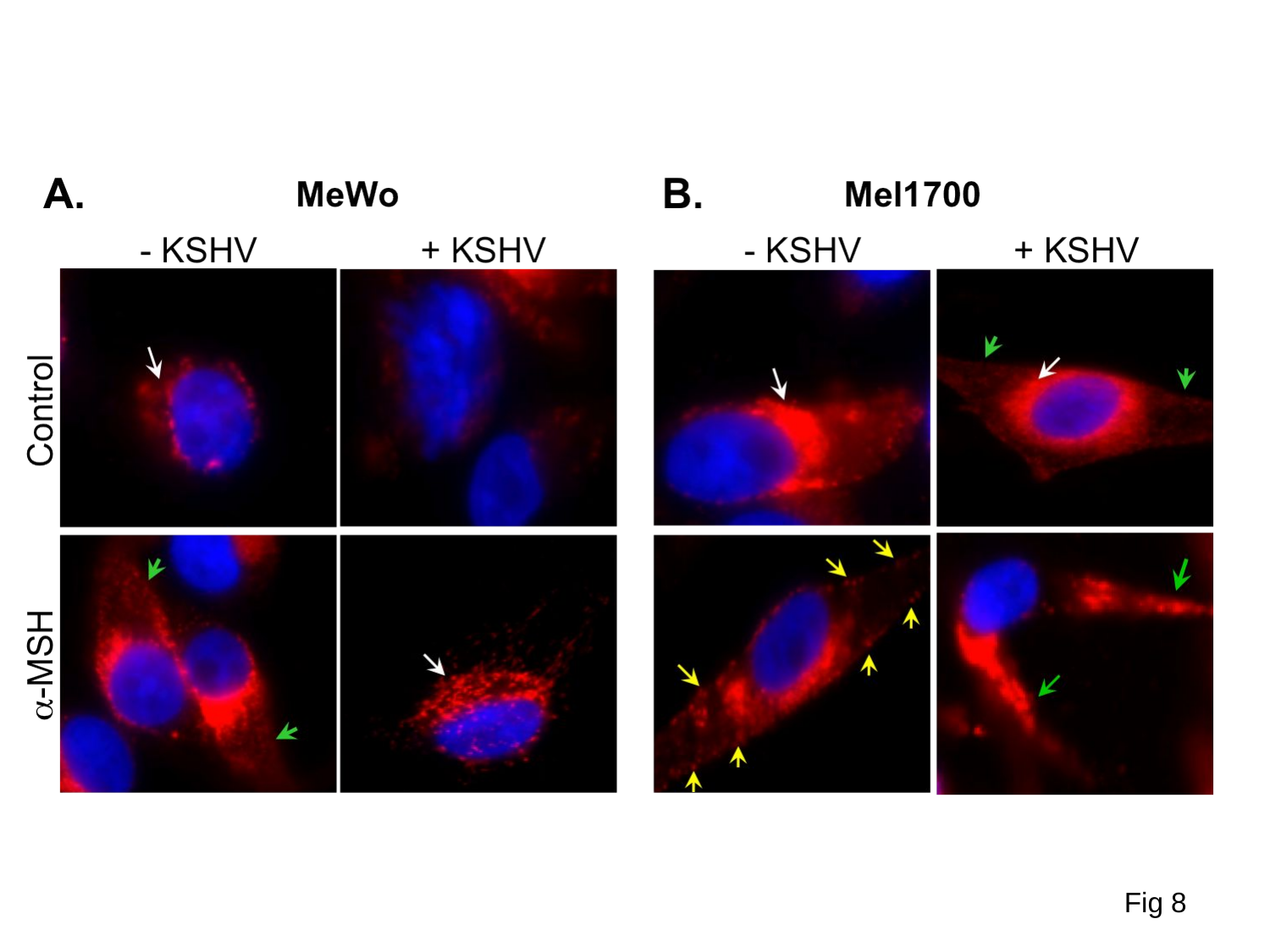

Fig 8

## Slide 9
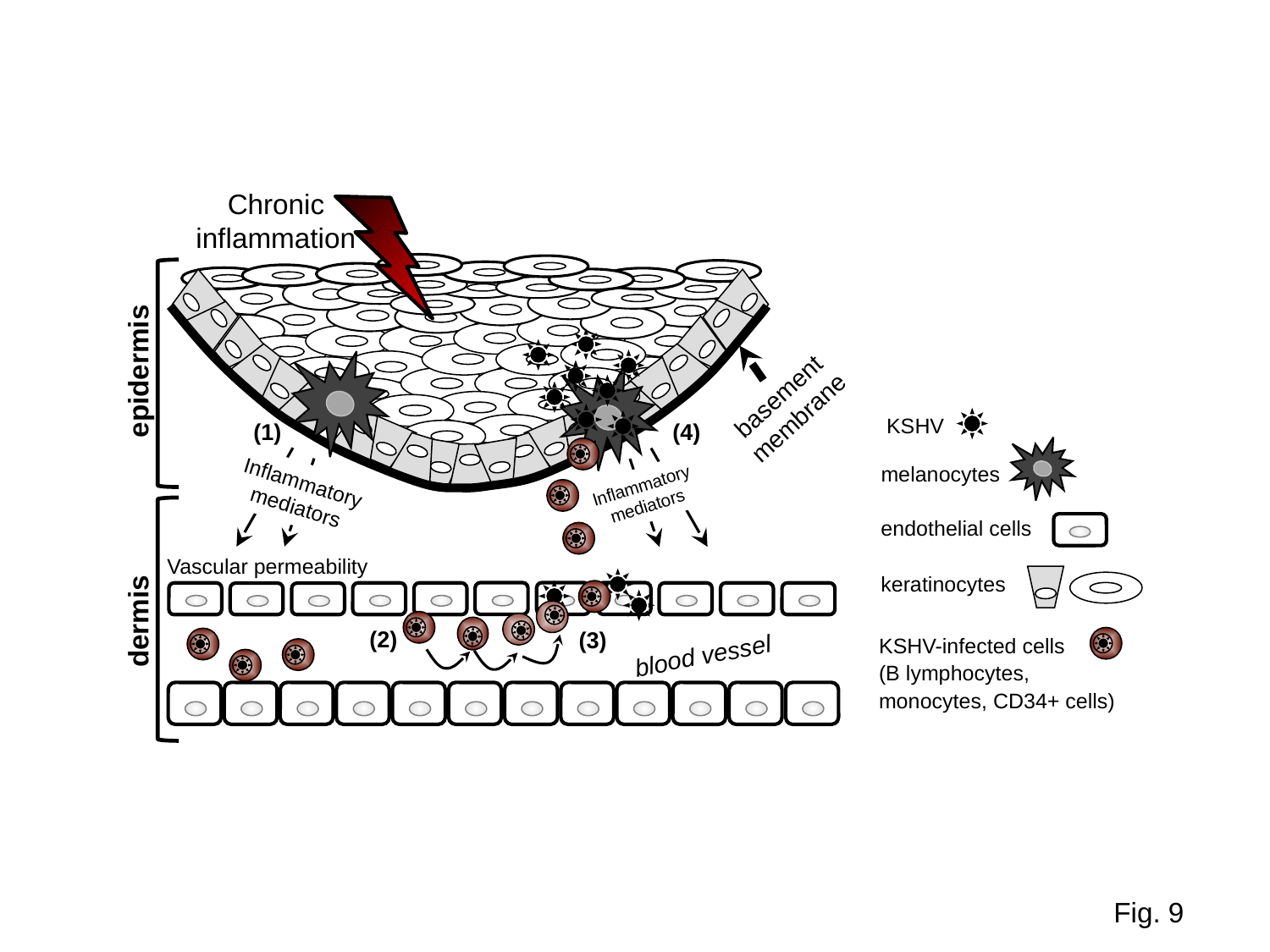

Chronic
inflammation
epidermis
basement
membrane
KSHV
(1)
(4)
melanocytes
Inflammatory
mediators
Inflammatory
mediators
endothelial cells
Vascular permeability
keratinocytes
dermis
(2)
(3)
KSHV-infected cells
(B lymphocytes, monocytes, CD34+ cells)
blood vessel
Fig. 9

## Slide 10
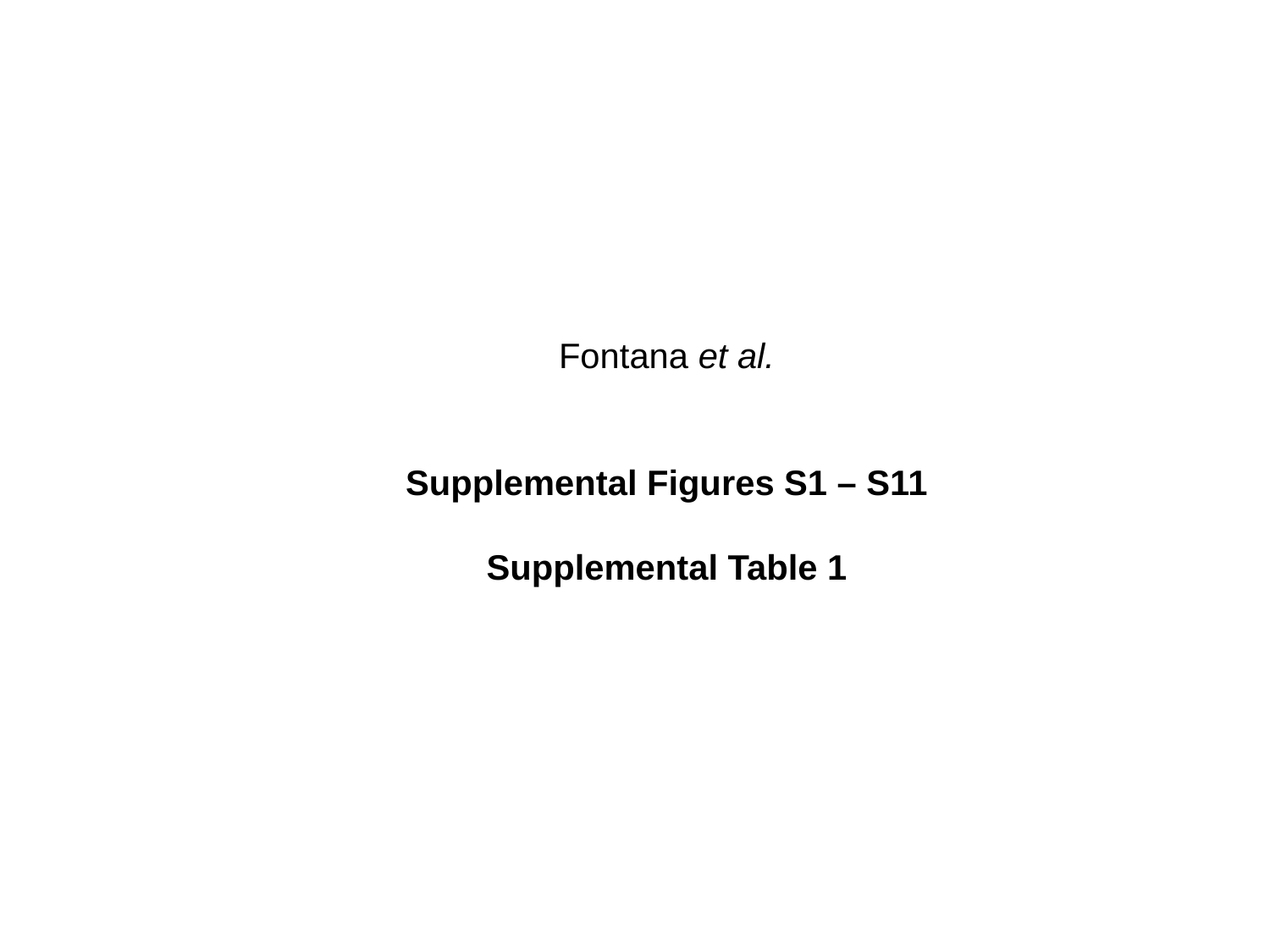

# Fontana et al. Supplemental Figures S1 – S11 Supplemental Table 1

## Slide 11
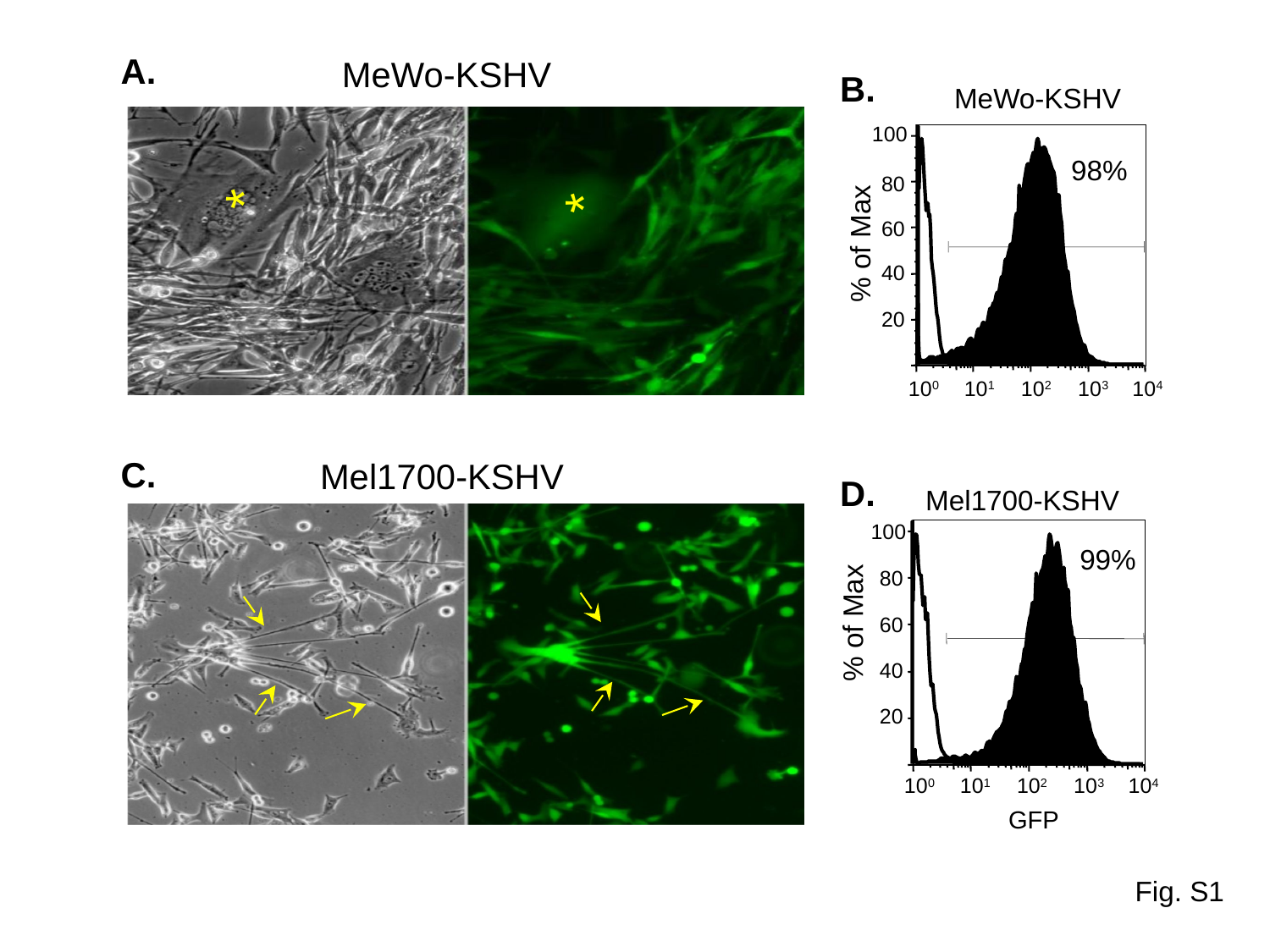

*
*
A.
MeWo-KSHV
B.
MeWo-KSHV
100
98%
80
60
40
20
100
101
102
103
104
% of Max
C.
Mel1700-KSHV
D.
Mel1700-KSHV
100
99%
80
60
40
20
101
102
103
104
100
% of Max
GFP
Fig. S1

## Slide 12
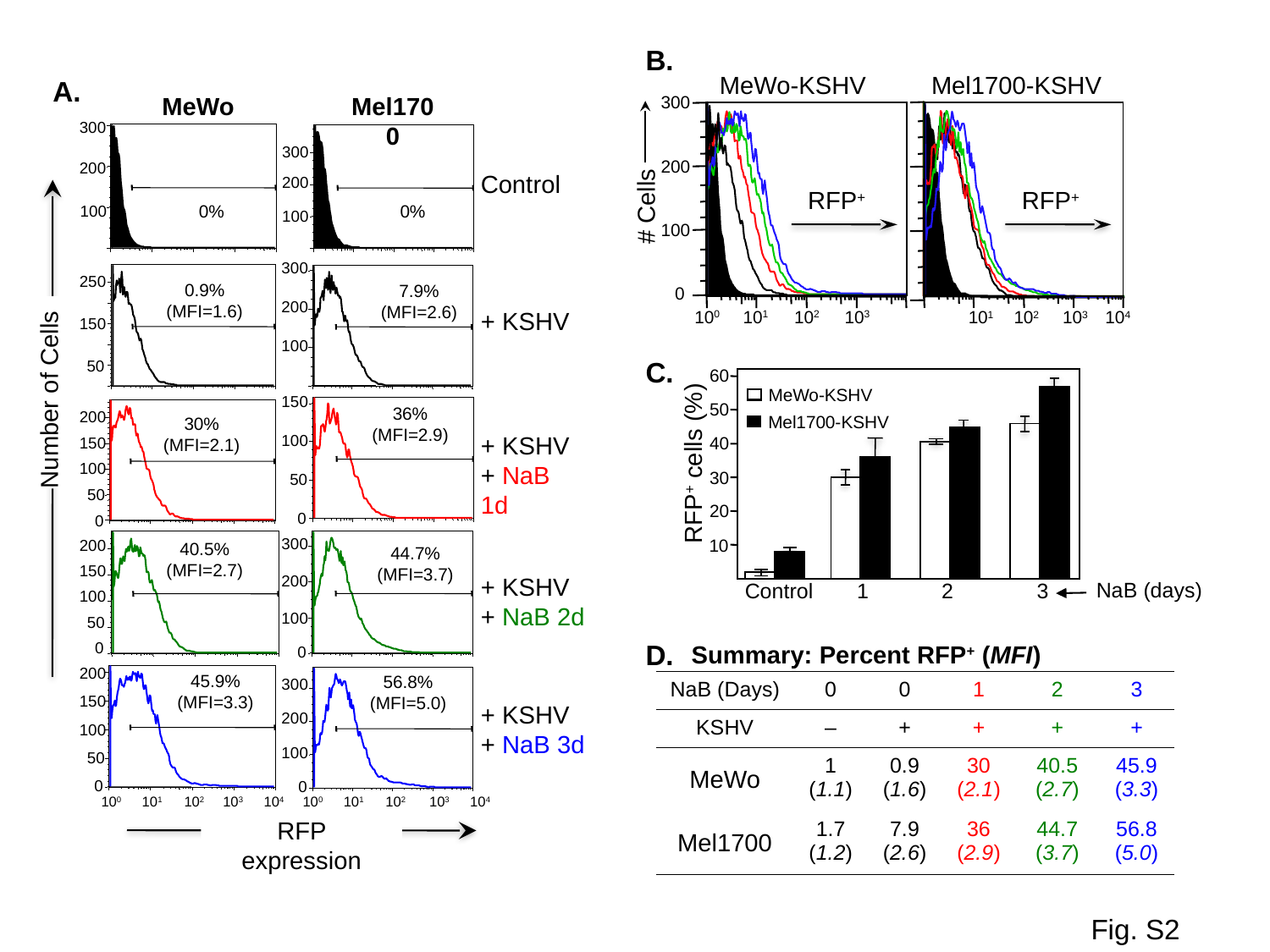

B.
MeWo-KSHV
Mel1700-KSHV
300
200
# Cells
RFP+
RFP+
100
0
100
101
102
103
101
102
103
104
A.
MeWo
Mel1700
300
0%
0%
300
200
100
200
Control
100
300
7.9%
(MFI=2.6)
200
100
250
150
50
0.9%
(MFI=1.6)
+ KSHV
Number of Cells
150
100
50
0
36%
(MFI=2.9)
200
150
100
50
0
30%
(MFI=2.1)
+ KSHV
+ NaB 1d
300
200
100
0
44.7%
(MFI=3.7)
200
150
100
50
0
40.5%
(MFI=2.7)
+ KSHV
+ NaB 2d
200
150
100
50
0
45.9%
(MFI=3.3)
56.8%
(MFI=5.0)
300
200
100
0
+ KSHV
+ NaB 3d
102
101
103
104
100
100
101
102
103
104
RFP expression
C.
60
MeWo-KSHV
50
Mel1700-KSHV
40
RFP+ cells (%)
30
20
10
NaB (days)
Control
1
2
3
D.
Summary: Percent RFP+ (MFI)
| NaB (Days) | 0 | 0 | 1 | 2 | 3 |
| --- | --- | --- | --- | --- | --- |
| KSHV | – | + | + | + | + |
| MeWo | 1 (1.1) | 0.9 (1.6) | 30 (2.1) | 40.5 (2.7) | 45.9 (3.3) |
| Mel1700 | 1.7 (1.2) | 7.9 (2.6) | 36 (2.9) | 44.7 (3.7) | 56.8 (5.0) |
Fig. S2

## Slide 13
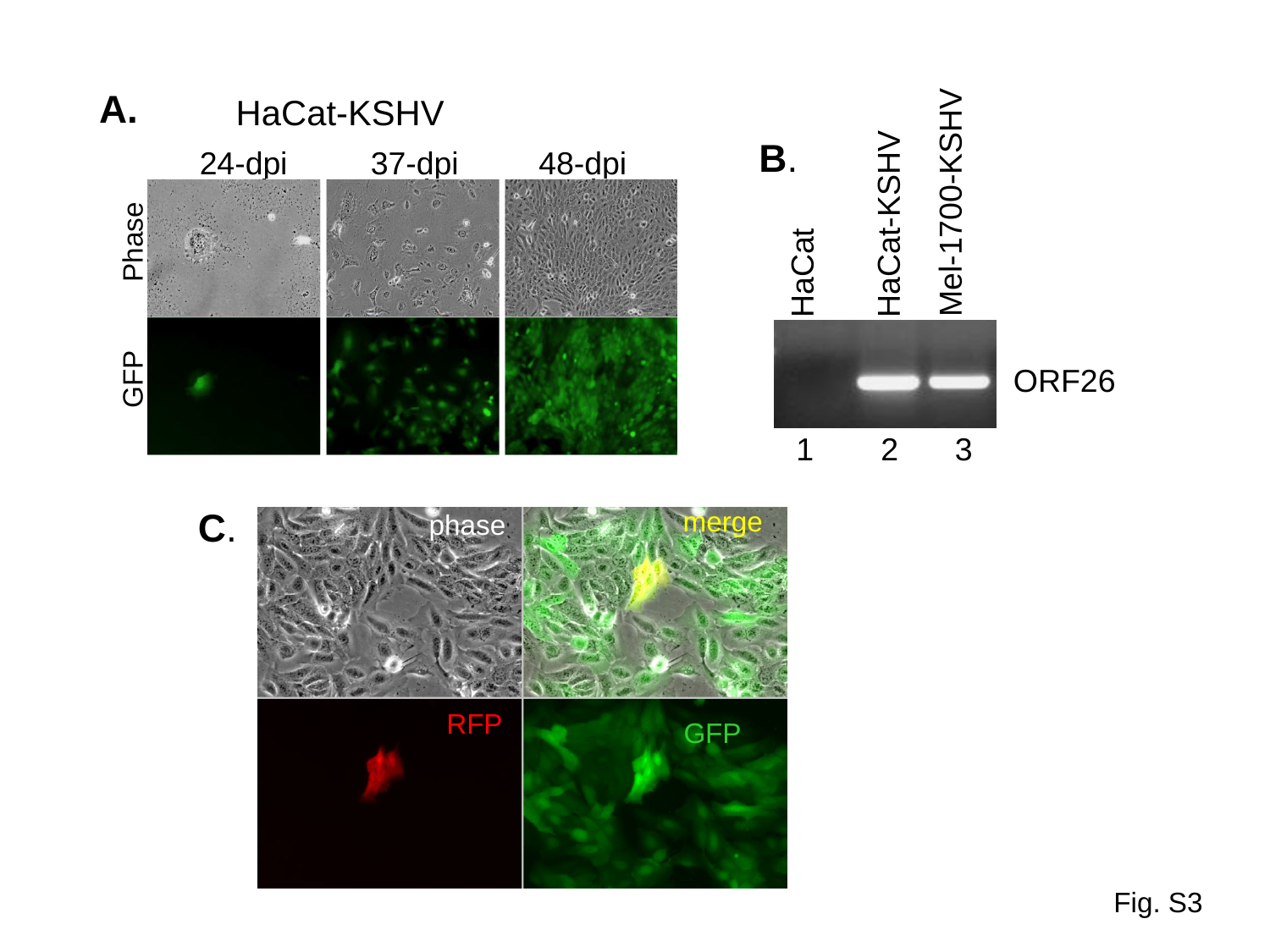

B.
Mel-1700-KSHV
HaCat-KSHV
HaCat
ORF26
1
2
3
A.
HaCat-KSHV
24-dpi
37-dpi
48-dpi
Phase
GFP
C.
merge
phase
RFP
GFP
Fig. S3

## Slide 14
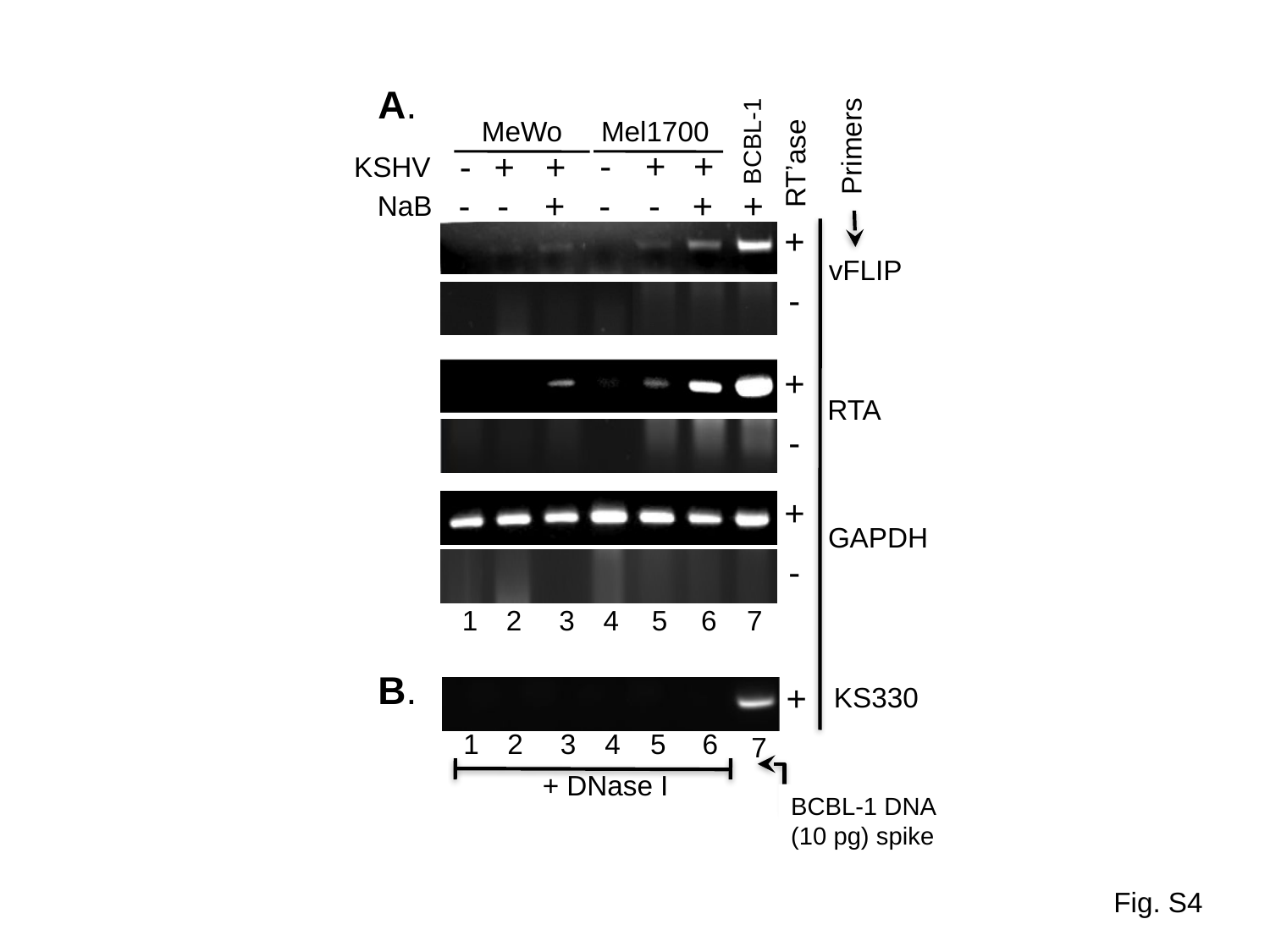

A.
MeWo
Mel1700
BCBL-1
Primers
RT’ase
KSHV
-
+
+
-
+
+
+
NaB
-
-
+
-
-
+
+
vFLIP
-
+
RTA
-
+
GAPDH
-
1
2
3
4
5
6
7
+
KS330
1
2
3
4
5
6
+ DNase I
BCBL-1 DNA (10 pg) spike
B.
7
Fig. S4

## Slide 15
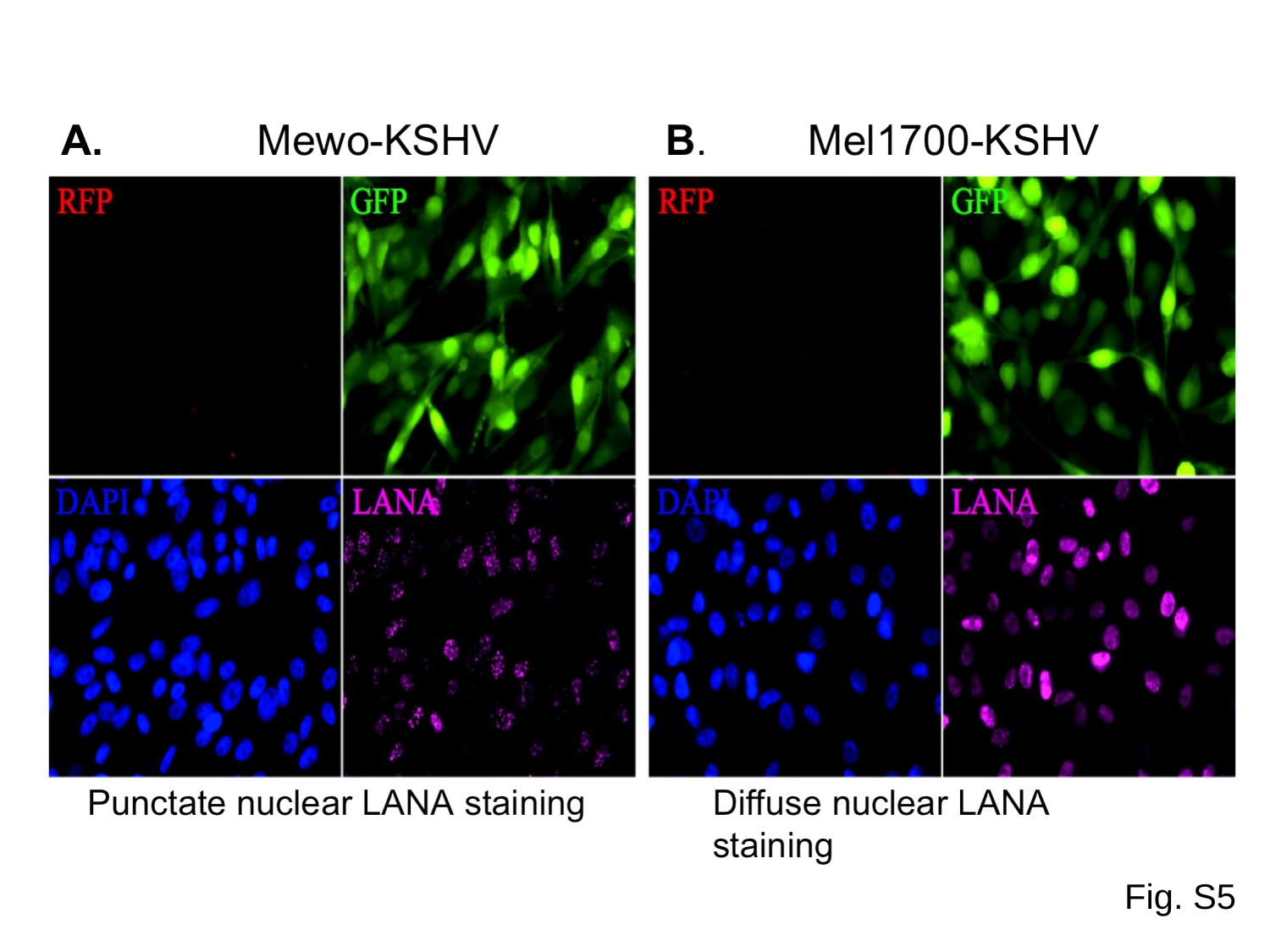

Fig. S5

## Slide 16
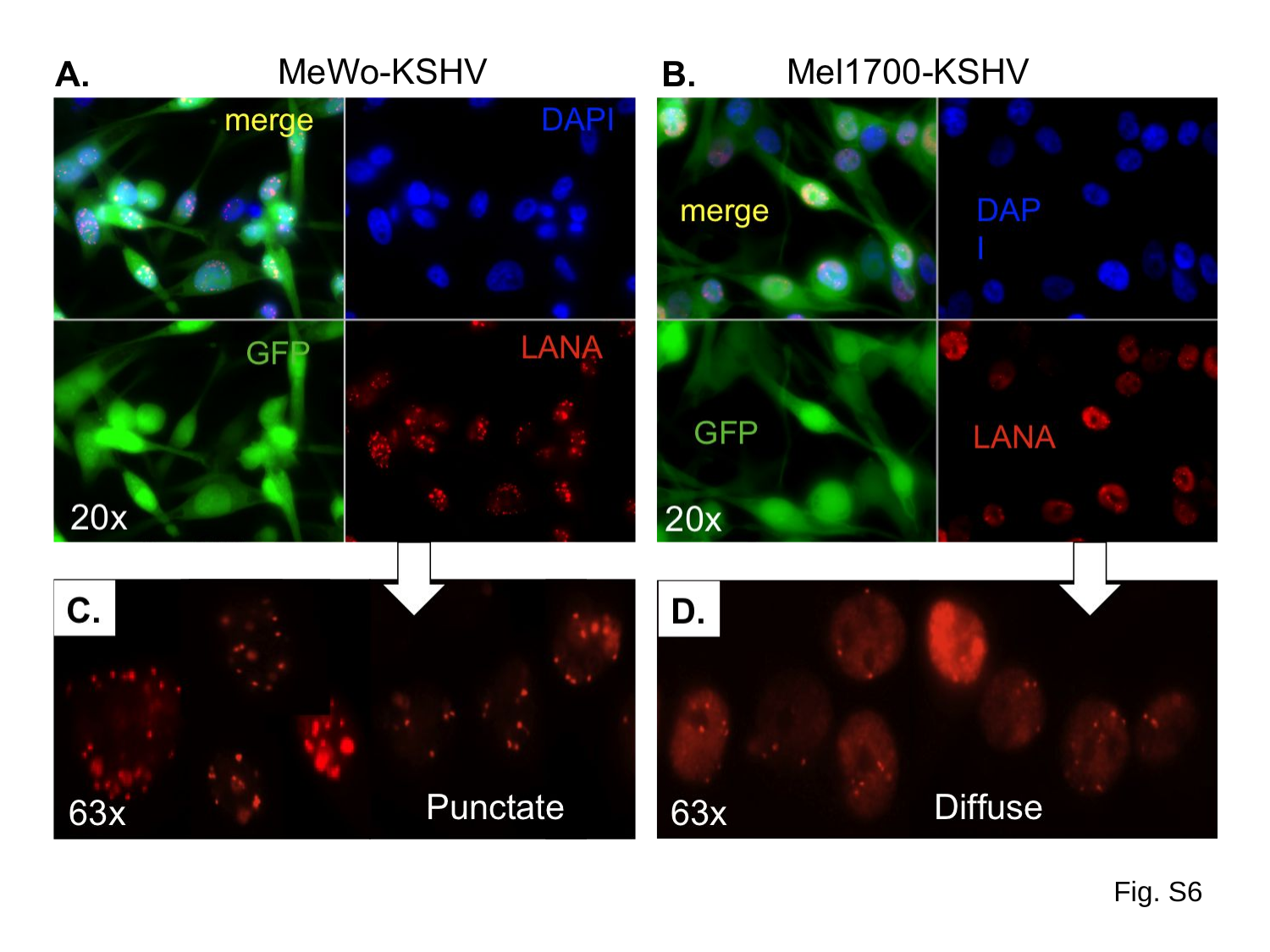

Fig. S6

## Slide 17
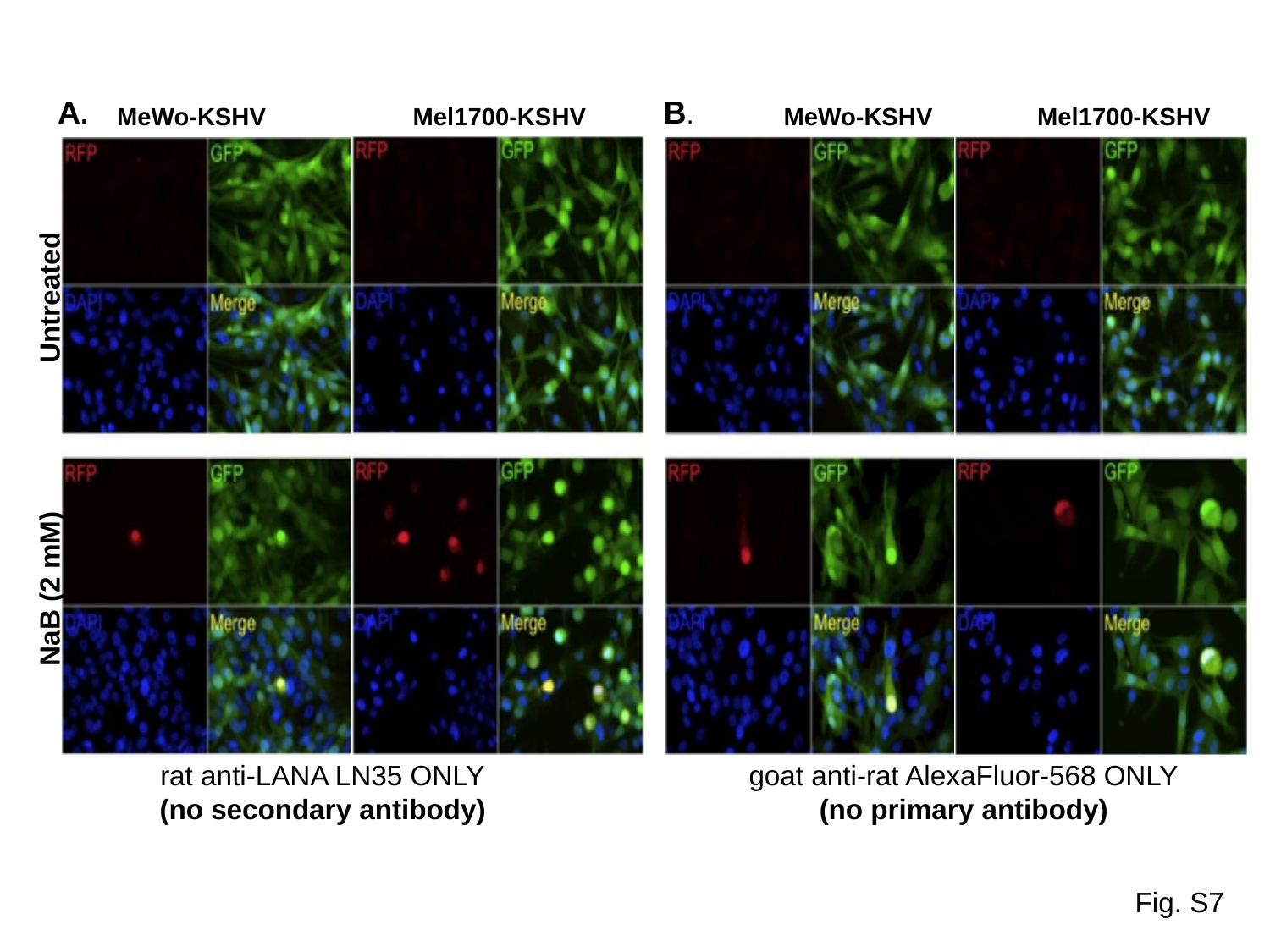

A.
B.
MeWo-KSHV
Mel1700-KSHV
MeWo-KSHV
Mel1700-KSHV
Untreated
NaB (2 mM)
rat anti-LANA LN35 ONLY
(no secondary antibody)
goat anti-rat AlexaFluor-568 ONLY
(no primary antibody)
Fig. S7

## Slide 18
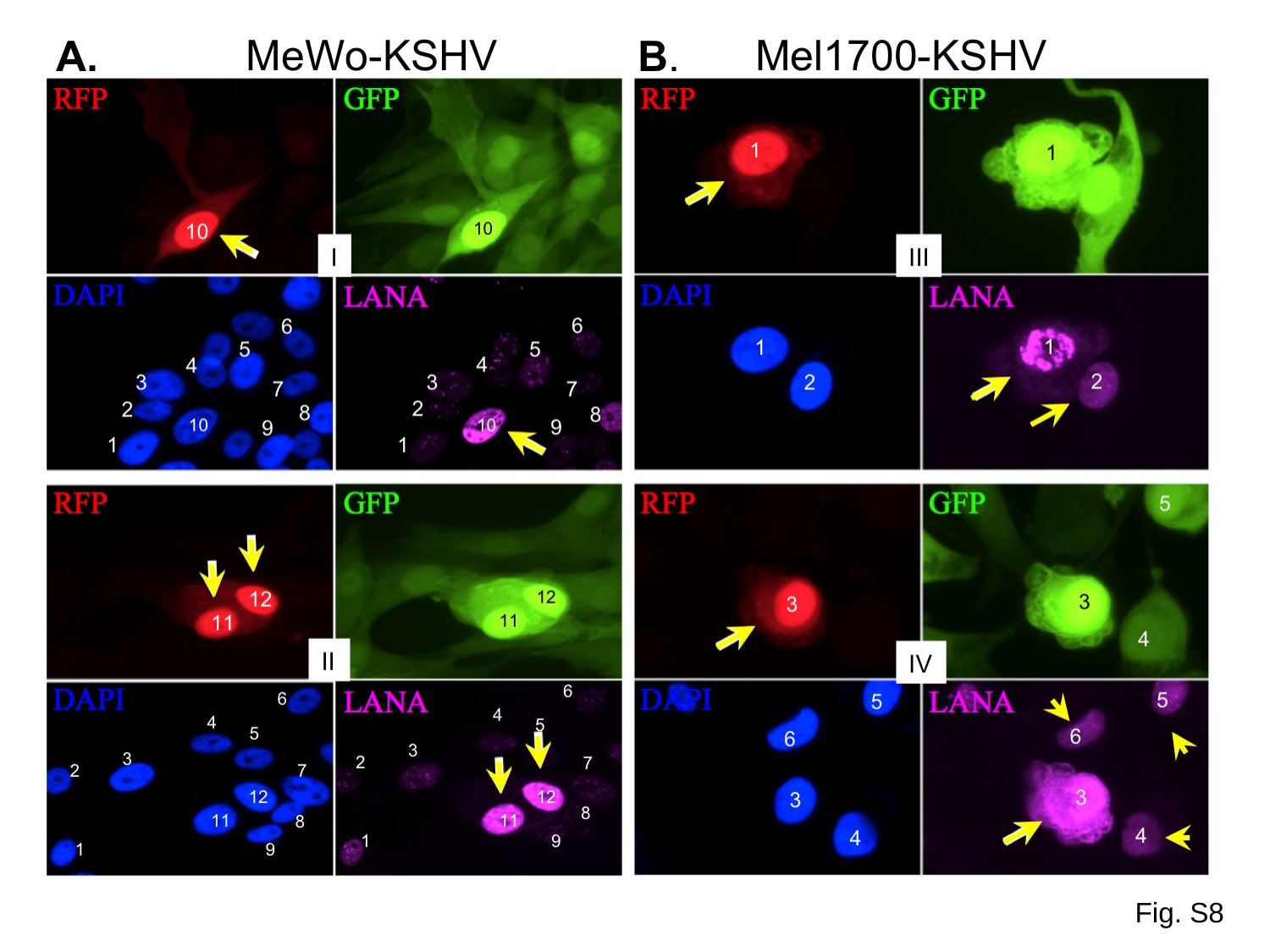

Fig. S8

## Slide 19
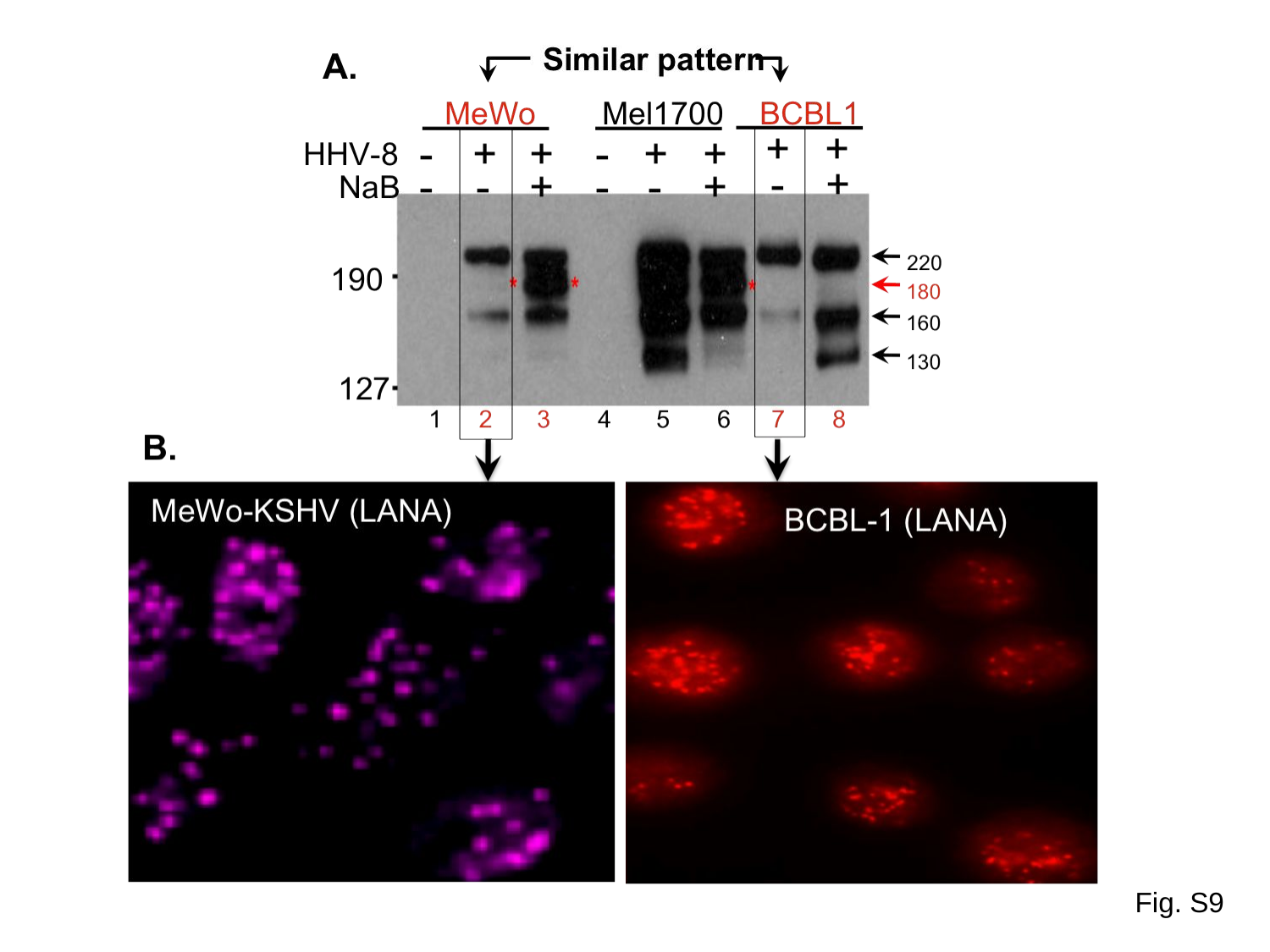

Fig. S9

## Slide 20
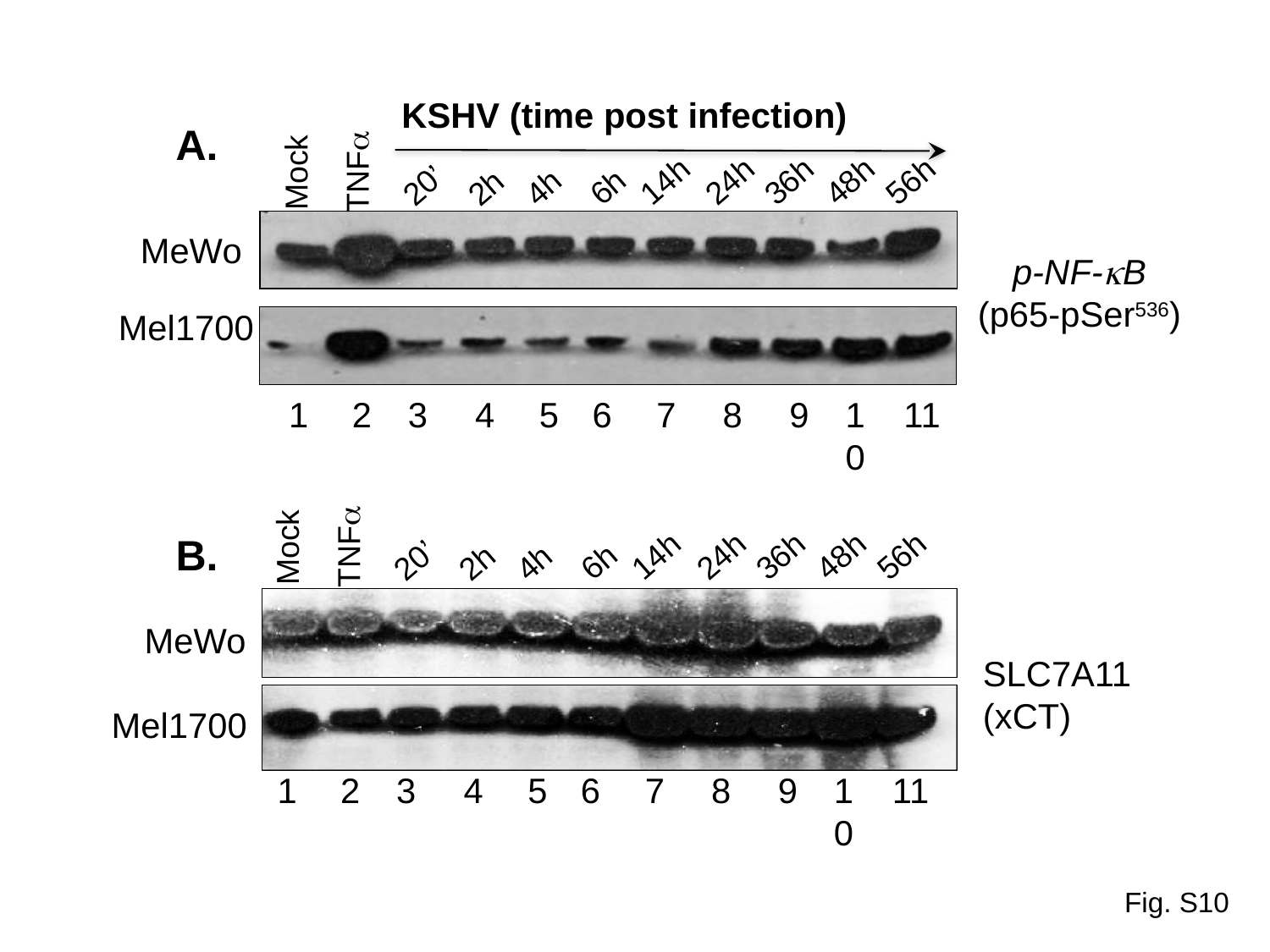

KSHV (time post infection)
TNFa
Mock
24h
36h
48h
56h
14h
6h
4h
20’
2h
p-NF-kB
(p65-pSer536)
TNFa
Mock
24h
36h
48h
56h
14h
6h
4h
20’
2h
SLC7A11
(xCT)
A.
MeWo
Mel1700
1
2
3
4
5
6
7
8
9
10
11
B.
MeWo
Mel1700
1
2
3
4
5
6
7
8
9
10
11
Fig. S10

## Slide 21
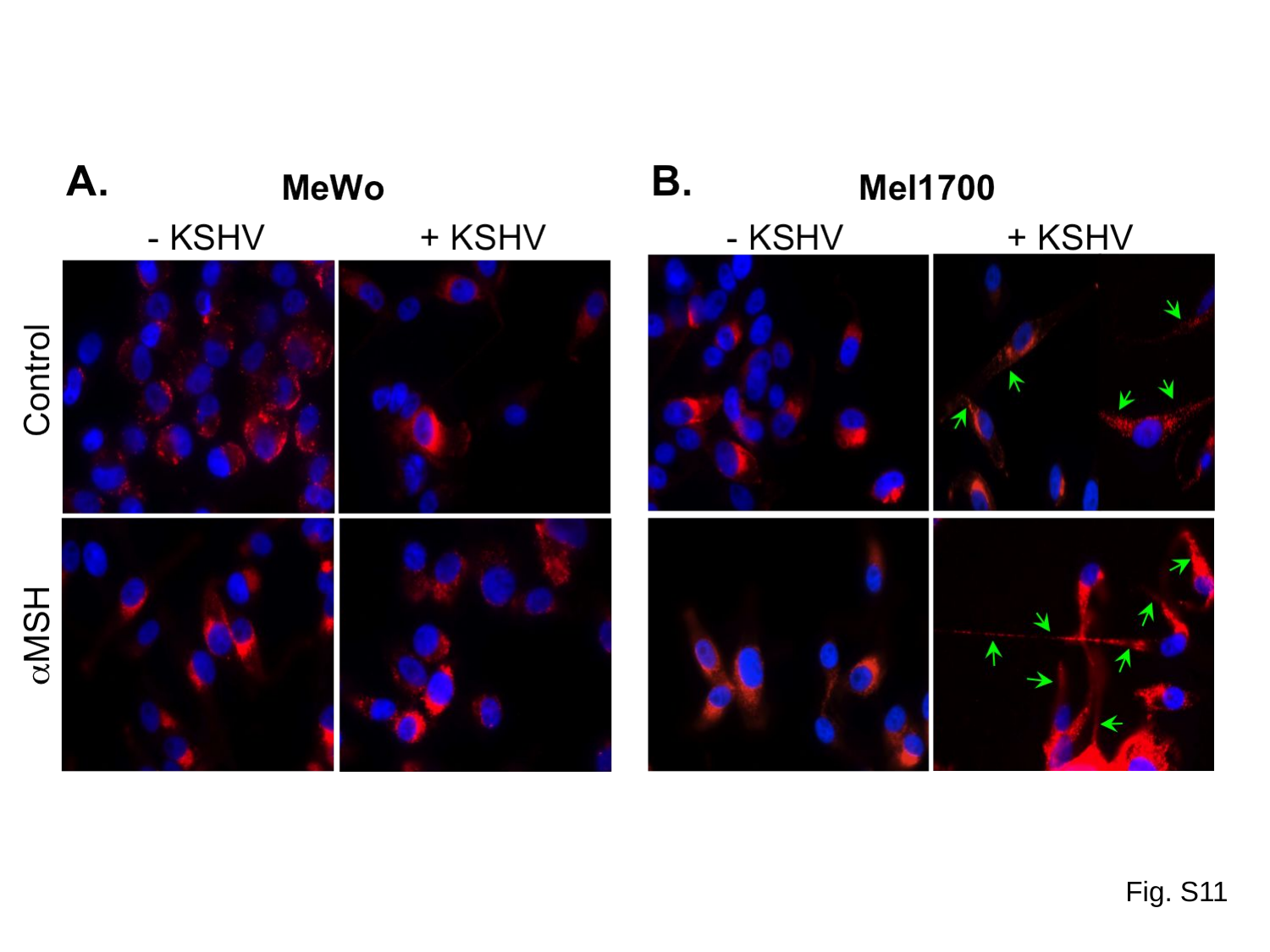

Fig. S11

## Slide 22
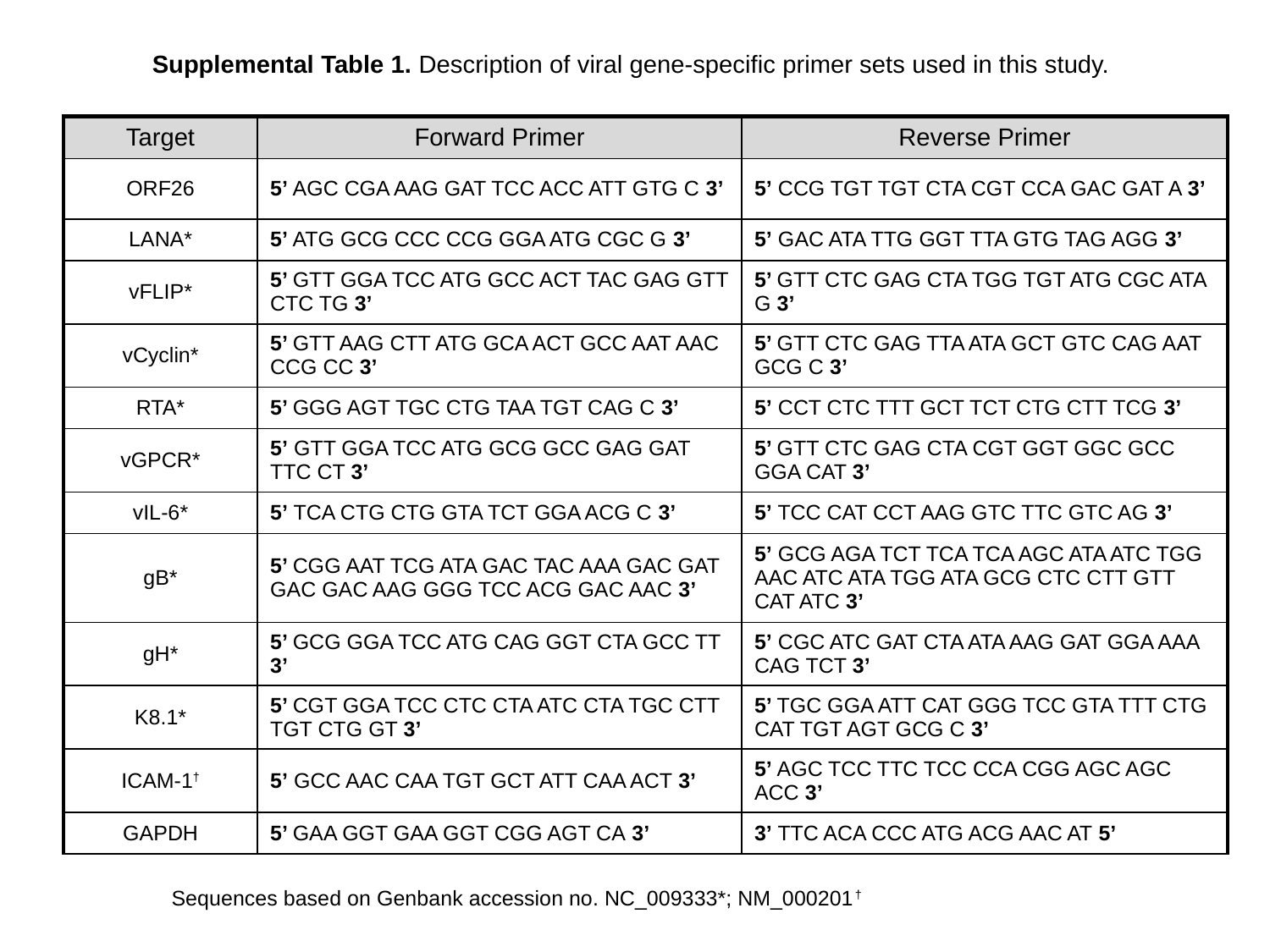

Supplemental Table 1. Description of viral gene-specific primer sets used in this study.
| Target | Forward Primer | Reverse Primer |
| --- | --- | --- |
| ORF26 | 5’ AGC CGA AAG GAT TCC ACC ATT GTG C 3’ | 5’ CCG TGT TGT CTA CGT CCA GAC GAT A 3’ |
| LANA\* | 5’ ATG GCG CCC CCG GGA ATG CGC G 3’ | 5’ GAC ATA TTG GGT TTA GTG TAG AGG 3’ |
| vFLIP\* | 5’ GTT GGA TCC ATG GCC ACT TAC GAG GTT CTC TG 3’ | 5’ GTT CTC GAG CTA TGG TGT ATG CGC ATA G 3’ |
| vCyclin\* | 5’ GTT AAG CTT ATG GCA ACT GCC AAT AAC CCG CC 3’ | 5’ GTT CTC GAG TTA ATA GCT GTC CAG AAT GCG C 3’ |
| RTA\* | 5’ GGG AGT TGC CTG TAA TGT CAG C 3’ | 5’ CCT CTC TTT GCT TCT CTG CTT TCG 3’ |
| vGPCR\* | 5’ GTT GGA TCC ATG GCG GCC GAG GAT TTC CT 3’ | 5’ GTT CTC GAG CTA CGT GGT GGC GCC GGA CAT 3’ |
| vIL-6\* | 5’ TCA CTG CTG GTA TCT GGA ACG C 3’ | 5’ TCC CAT CCT AAG GTC TTC GTC AG 3’ |
| gB\* | 5’ CGG AAT TCG ATA GAC TAC AAA GAC GAT GAC GAC AAG GGG TCC ACG GAC AAC 3’ | 5’ GCG AGA TCT TCA TCA AGC ATA ATC TGG AAC ATC ATA TGG ATA GCG CTC CTT GTT CAT ATC 3’ |
| gH\* | 5’ GCG GGA TCC ATG CAG GGT CTA GCC TT 3’ | 5’ CGC ATC GAT CTA ATA AAG GAT GGA AAA CAG TCT 3’ |
| K8.1\* | 5’ CGT GGA TCC CTC CTA ATC CTA TGC CTT TGT CTG GT 3’ | 5’ TGC GGA ATT CAT GGG TCC GTA TTT CTG CAT TGT AGT GCG C 3’ |
| ICAM-1† | 5’ GCC AAC CAA TGT GCT ATT CAA ACT 3’ | 5’ AGC TCC TTC TCC CCA CGG AGC AGC ACC 3’ |
| GAPDH | 5’ GAA GGT GAA GGT CGG AGT CA 3’ | 3’ TTC ACA CCC ATG ACG AAC AT 5’ |
Sequences based on Genbank accession no. NC_009333*; NM_000201†
